# Post-marketing safety surveillance of tetanus, diphtheria, and acellular pertussis (Tdap) vaccine: a disproportionality analysis of the vaccine adverse event reporting system (2015–2025)

**DOI:** 10.3389/fcimb.2026.1831548

**Published:** 2026-09-08

**Authors:** Wenyan Song, Hongjun Chen, Meiyan Wang, Zhenye Liang

**Affiliations:** 1 Department of Pharmacy, Taizhou First People’s Hospital, Taizhou, China; 2 Department of Gastroenterology, Taizhou First People’s Hospital, Taizhou, China; 3 Department of Respiratory Medicine, Taizhou First People’s Hospital, Taizhou, China

**Keywords:** adverse events, disproportionality analysis, pharmacovigilance, Tdap vaccine, vaccine adverse event reporting system (VAERS), vaccine safety

## Abstract

**Background:**

The tetanus, diphtheria, and acellular pertussis (Tdap) vaccine is routinely recommended for adolescents, adults, and pregnant individuals. Ongoing post-marketing surveillance is critical to monitor its safety profile and detect rare or unexpected adverse events (AEs) following immunization. This study aims to comprehensively analyze Tdap-related AEs reported to the Vaccine Adverse Event Reporting System (VAERS) over the past decade, with a focus on safety profiles in specific subpopulations.

**Methods:**

We retrospectively analyzed US AE reports related to Tdap vaccination from VAERS for the period 2015 to May 2025. Data mining was conducted using four disproportionality algorithms: Reporting Odds Ratio (ROR), Proportional Reporting Ratio (PRR), Bayesian Confidence Propagation Neural Network (BCPNN), and Multi-Item Gamma Poisson Shrinkage (MGPS). The False Discovery Rate (FDR) was controlled at 5% to minimize false positives. Subgroup analyses focused on adolescents (aged 10–18 years), older adults (aged ≥65 years), pregnant individuals, and sex-based subgroups (males vs. non-pregnant females). Time of onset was characterized using Weibull distribution analysis.

**Results:**

A total of 20,128 AE reports were identified, with 95.80% classified as non-serious. Injection site erythema, injection site swelling, and erythema were the most frequently reported positive signals. The majority of AEs (70.31%) occurred within one day of vaccination, showing an “early failure” pattern in Weibull analysis. Tdap did not exhibit any disproportionate accumulation of unexpected AEs among older adults. Among 744 pregnancy-related reports, no new or unexpected safety signals related to maternal or fetal outcomes were detected. Notably, the adolescent subgroup showed a significant signal for opisthotonus (ROR = 8.55). In non-pregnant females, bursal fluid accumulation (ROR = 21.22) and radiculitis brachial (ROR = 14.42) were disproportionately elevated, whereas no such signals were found in males.

**Conclusions:**

This 10-year surveillance study revealed no unexpected signals for Tdap in the overall population, older adults, males, or pregnant women. While most reports were non-serious, the signals of radiculitis brachial in non-pregnant females generate hypotheses that require formal testing in active surveillance systems. Our findings are generally consistent with the current Tdap vaccination recommendations and highlight the importance of ongoing post-marketing surveillance.

## 1 Introduction

Pertussis is a highly contagious acute respiratory infection caused by Bordetella pertussis (Decker and Edwards, 2021). It exhibits periodic epidemics every 3 to 5 years and has recently re-emerged in numerous countries and regions (Christie, 2025). According to the World Health Organization (WHO), more than 939,178 cases of pertussis were reported globally in 2024 (Vargas-Zambrano et al., 2026). Pertussis can occur at any age. Although infants remain at the highest risk for severe disease and death, epidemiological trends indicate that the peak incidence of pertussis has shifted from infants to adolescents and adults (Skoff et al., 2015). These individuals are frequently underdiagnosed due to atypical presentations, making them a main sources of transmission to infants. Multiple studies have further confirmed that siblings and mothers are the most common source of pertussis infection in newborns (Skoff et al., 2015). Moreover, older adults are more susceptible to pertussis due to immunosenescence and comorbidities, leading to higher rates of hospitalization and mortality compared with younger individuals (Kardos et al., 2024). Increased reporting of pertussis in adolescents and adults can be attributed to multiple factors, most notably the waning immunity following childhood primary vaccination.

The tetanus toxoid, reduced diphtheria toxoid, and acellular pertussis vaccine (Tdap) has therefore become an essential component of pertussis prevention across multiple populations (Martinón-Torres et al., 2018). In 2005, the Advisory Committee on Immunization Practices (ACIP) recommended Tdap for adolescents and adults (Broder et al., 2006; Kretsinger et al., 2006). The vaccination recommendation was expanded to adults aged 65 years and older in 2012 (Centers for Disease Control and Prevention (CDC), 2012). In the same year, ACIP further recommended that pregnant individuals receive a dose of Tdap during each pregnancy, regardless of prior vaccination history (Centers for Disease Control and Prevention (CDC), 2013). Maternal Tdap vaccination has become a cornerstone strategy for protecting newborns through passive transplacental antibody transfer. Currently, two Tdap vaccines have been licensed in the U.S.: Adacel (Sanofi Pasteur) and Boostrix (GlaxoSmithKline Biologicals).

Tdap and diphtheria, tetanus, and acellular pertussis (DTaP) vaccines are both acellular pertussis-containing combination vaccines with related antigenic components and similar mechanisms of action (Liang et al., 2018). The extensive safety data on DTaP provides useful historical context for understanding pertussis-containing vaccine safety more broadly. Studies of DTaP have consistently shown that local reactions and transient systemic symptoms are most commonly reported, such as injection site erythema, injection site swelling and pyrexia, whereas serious adverse events (AEs) are uncommon (Liu et al., 2026; Moro et al., 2018a; Patterson et al., 2018). Compared with Tdap, DTaP is primarily administered to infants and children younger than 7 years and contains higher per-dose antigen content of diphtheria and pertussis components. Accordingly, DTaP safety data serve as an informative reference but should not be directly extrapolated to Tdap, given the differences in antigen content, immunization roles and target populations. Therefore, independent and comprehensive safety evaluations specifically for Tdap remain essential.

Evidence accumulated from clinical trials, post-licensure observational studies, and systematic reviews has demonstrated that Tdap is well tolerated in adolescents, adults, older adults, and pregnant women (McMillan et al., 2017; Naleway et al., 2025; Haber et al., 2020; Park et al., 2019). The most common AEs include injection site pain, erythema, headache, and fatigue. Despite the substantial evidence supporting the overall safety of Tdap, concerns persist regarding specific outcomes. Among pregnant women, numerous large-scale observational studies, including analyses based on the Vaccine Safety Datalink (VSD), have found no consistent association between Tdap vaccination and most serious maternal or neonatal adverse outcomes (Baggs et al., 2011; Florea et al., 2023; Greenberg et al., 2023). However, the evidence base for certain outcomes, such as chorioamnionitis, remains inconsistent (Moro et al., 2015; Vygen-Bonnet et al., 2020; Greenberg et al., 2023). In addition, concerns persist regarding rare neurological adverse events following Tdap vaccination, and the evidence for causal associations remains limited (Baxter et al., 2016; Stratton, 1994).

Notably, several limitations persist in the existing evidence on Tdap safety. Emerging evidence suggests that adverse events following immunization may differ by sex, underscoring the urgent need for sex-stratified safety assessment to enable more precise risk characterization (Duijster et al., 2023; Zhou et al., 2025). However, sex-specific evidence on Tdap safety remains limited. And most prior studies have focused on a single target population, limiting direct comparison of safety reporting patterns across key subgroups under a unified analytic framework. Additionally, some studies have been constrained by limited sample sizes, hindering the assessment of rare or potentially serious AEs. Moreover, as Tdap is increasingly co-administered with other vaccines and administered repeatedly as a booster, its real-world utilization has become more complex. These evolving immunization practices may alter the Tdap’s safety profile, warranting updated post-marketing safety studies for re-evaluation.

The Vaccine Adverse Event Reporting System (VAERS) is a widely used passive surveillance system for monitoring vaccine safety (Singleton, 1999). It enables near real-time identification of potential vaccine safety concerns and detection of rare vaccine adverse events (Iskander et al., 2004). In this study, we evaluated Tdap-associated AEs reported to the VAERS over the past decade, and conducted a comprehensive disproportionality analysis in the overall population and key subgroups. Data mining was performed using four signal detection algorithms with cross-validation, and the false discovery rate (FDR) was applied to control for multiple testing. This study aims to update the real-world safety profile of the Tdap vaccine and provide evidence to inform clinical vaccination strategies.

## 2 Materials and methods

### 2.1 Data source

This study utilized data from the VAERS. VAERS, jointly administered by the Centers for Disease Control and Prevention (CDC) and the Food and Drug Administration (FDA), is a nationwide passive vaccine safety surveillance system that accepts reports of adverse events following vaccination (Shimabukuro et al., 2015). The principal advantages of VAERS lie in its comprehensive national scope and its capacity to accept reports in near real-time, which is critical for the timely identification of rare or unexpected AEs. These attributes position VAERS as an initial signal detection tool for generating safety hypotheses. However, VAERS lacks denominator data and is inherently limited by reporting biases, which does not permit causal inference. Therefore, its findings require further validation in active surveillance systems or well-designed epidemiologic studies. The VAERS report form collects information on patient demographics, vaccination details, and adverse event descriptions. Signs and symptoms of AEs were coded using the Preferred Terms (PTs) and categorized into System Organ Class (SOC), as defined by Medical Dictionary for Regulatory Activities (MedDRA, version 28.1) (Brown, 2004). Each VAERS report may be assigned one or more PTs.

### 2.2 Study population and report identification

#### 2.2.1 Identification of Tdap-related reports

We searched the VAERS database for US reports related to Tdap vaccination from January 1, 2015, to May 31, 2025. Records corresponding to Boostrix or Adacel were identified by vaccine name and extracted. Reports flagged as foreign or duplicate reports were excluded.

#### 2.2.2 Identification of pregnancy-related reports

To search for pregnancy reports, the automated search strategy was utilized, following Moro et al.’s methodology (Moro et al., 2018b):

- SOC-based search: Reports including any of the following MedDRA SOC: “Pregnancy, Puerperium, and Perinatal Conditions” and “Congenital, Familial and Genetic Disorders”;
- PT-based search: Reports containing any of the following MedDRA PTs: “Drug exposure during pregnancy”, “Maternal exposure during pregnancy”, and “Exposure during pregnancy”;
- Free-text search: a text string search for the term “preg” in the report, with instructions to exclude mentions of “not pregnant”, “non-pregnant”, “non pregnant”, “nonpregnant”, and “no preg”.

Following further verification by manually screening the free-text fields (the SYMPTOM_TEXT, CUR_ILL, and HISTORY fields), we excluded reports that indicated the vaccinee was not pregnant or had received Tdap before the last menstrual period. Two clinicians independently reviewed these free-text entries, and any discrepancies were resolved through discussion.

### 2.3 Descriptive analysis

#### 2.3.1 Descriptive analysis of general reports

Based on the collected reports, descriptive statistics were performed to classify AE reports of Tdap by sex, age, onset time, clinical outcome, and seriousness. The onset time was defined as the time between the date of vaccination and the date of adverse event occurrence. Reports were classified as serious if any of the following outcomes are reported: death, life-threatening illness, hospitalization, prolongation of existing hospitalization, or permanent disability. All data were extracted from the structured fields of the database.

#### 2.3.2 Descriptive analysis of pregnancy reports

Descriptive analyses of pregnancy reports included maternal age, trimester of pregnancy at time of vaccination, onset time, outcomes, seriousness, and concomitant vaccines. The gestational age at vaccination was estimated using the last menstrual period (LMP), estimated delivery date (EDD), or estimated date of conception as reported in the VAERS report and medical records. When such information was unavailable, gestational age was determined based on clinical assessment by healthcare professionals as stated or the earliest available ultrasound assessment. Trimester was defined as first (0–13 weeks), second (14–27 weeks), or third (≥28 weeks).

### 2.4 Disproportionality analysis and signal detection

Disproportionality analysis was employed as the primary data mining method for detecting signals of adverse events. In this study, we applied four algorithms for signal detection: Reporting odds ratio (ROR), proportional reporting ratio (PRR), Bayesian confidence propagation neural network (BCPNN), and Multi-item gamma Poisson shrinker (MGPS). The PRR has high specificity, while the ROR is less susceptible to reporting bias for low-frequency events (Evans et al., 2001; Rothman et al., 2004). BCPNN performs well in integrating data from multiple sources and cross-validation, whereas MGPS offers enhanced signal detection for rare events compared to BCPNN (Bate et al., 1998; Kubota et al., 2004). To ensure robustness of signal detection, a signal was classified as positive only when all four algorithms simultaneously indicated a positive result. Disproportionality analyses were performed using standardized 2×2 contingency tables (**Supplementary Table 1**), with consistent computational formulas and predefined statistical thresholds as provided in **Supplementary Table 2**. Analyses were performed for the overall dataset as well as for predefined subgroups according to age, sex, and pregnancy status.

The Benjamini-Hochberg procedure was applied separately within the overall dataset and within each subgroup analysis to control the within-analysis FDR at 5% (p-adjust < 0.05). This approach does not control the overall type I error rate across all subgroup analyses combined, and subgroup-specific findings should therefore be interpreted as exploratory.

### 2.5 Weibull distribution analysis

The Weibull distribution analysis was applied to assess the temporal relationship between vaccination and the onset of adverse events. The scale parameter α determines the spread of the distribution of time to AE onset, with larger values indicating a wider distribution. The shape parameter β describes how the risk of AE onset changes over time (Sauzet et al., 2013). The time-to-onset reporting patterns were categorized based on the shape parameter β:

- Early failure pattern: β < 1 (with the upper bound of the 95% confidence interval (CI) < 1) signifying that most AEs occur shortly after vaccination;
- Random failure pattern: β ≈ 1 (with the 95% CI including 1) indicating the occurrence of AEs is independent of time;
- Wear-out failure pattern: β > 1 (with the lower bound of the 95% CI > 1) suggesting delayed onset of most AEs.

### 2.6 Sensitivity analysis

#### 2.6.1 Complete-case sensitivity analysis

To assess the potential impact of missing or unknown data, we conducted a sensitivity analysis by excluding reports with key variables (age, sex, time to onset) recorded as “Unknown”. We then repeated the disproportionality analysis, and the resulting RORs and 95% CIs were compared with those from the primary analysis.

#### 2.6.2 Case-deletion sensitivity analysis

For subgroup signals based on small numbers of reports, we conducted a sequential case-deletion analysis in which one target-case report was removed at a time and the ROR with its 95% CI was recalculated after each deletion. This process continued until the signal no longer met the prespecified significance criterion.

#### 2.6.3 Single-vaccine Tdap sensitivity analysis

To assess confounding by concomitant vaccination, we repeated the disproportionality analysis in reports with Tdap administered alone, applying identical signal criteria as the primary analysis. The resulting RORs and 95% CIs were compared with the main findings to evaluate the impact of co-administration on signal detection.

#### 2.6.4 Restricted time-window disproportionality analysis

To assess the robustness of the primary findings, we performed restricted time-window disproportionality analysis for radiculitis brachial. This analysis was restricted to reports with symptom onset within the pre-specified risk window. The risk window for radiculitis brachial onset was defined as 0 to 28 days. Previous studies have shown that the majority of radiculitis brachial cases following vaccination occur within this time window, consistent with the known pathophysiological mechanisms (Stratton, 1994; Van Alfen and Van Engelen, 2006).

### 2.7 Reporting guidance

The reporting of this study was informed by the STROBE statement and the RECORD recommendations, where applicable to pharmacovigilance analyses based on routinely collected spontaneous reporting data.

## 3 Results

### 3.1 Descriptive analysis

**Figure 1:**
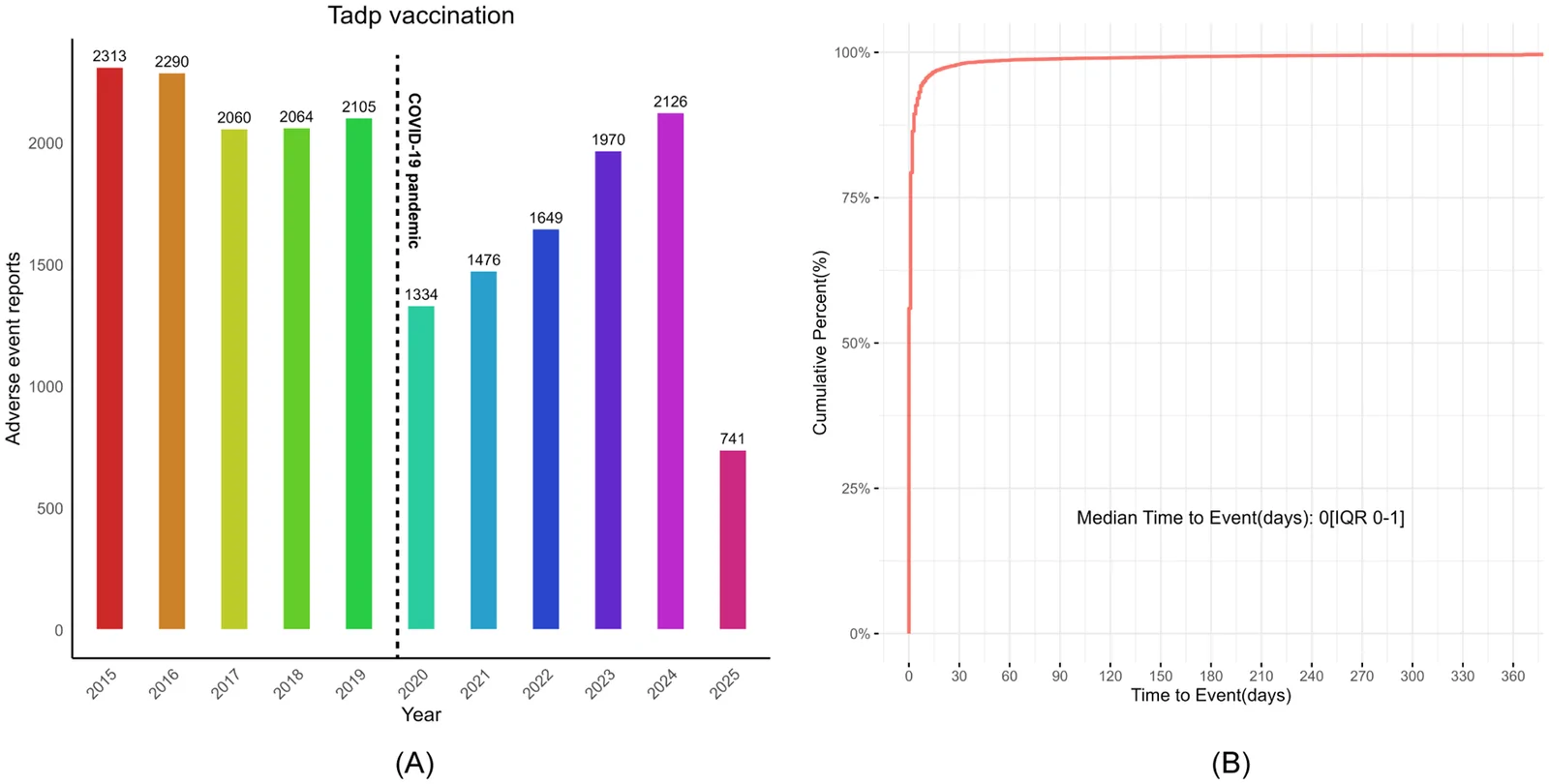
**(A)** Annual trends of adverse event reports related to Tdap from 2015 to 2025. Tdap, tetanus, diphtheria, and acellular pertussis. **(B)** Onset time distribution of adverse events.

A total of 20,128 AE reports related to Tdap were identified in this study, including 74,393 adverse events. The number of annual reports showed no significant fluctuation between 2015 and 2019, but declined markedly in 2020 (n = 1,334) and then gradually increased thereafter (**Figure 1A**). This trend likely reflected the impact of the COVID-19 pandemic. Among all reports, 60.61% were from females and 35.97% from males (**Table 1**). In terms of age distribution, the 19–64 years age group accounted for 47.66%, followed by those aged 10 to 18 years(31.75%). Reports from individuals aged 65 and older were relatively uncommon, comprising less than 10% of the total. Analysis of vaccine exposure indicated that Tdap was administered alone in 46.36% of reports, while 53.64% involved co-administration with other vaccines. Regarding clinical seriousness, most reports were categorized as non-serious (95.80%), while serious reports constituted 4.20%. Regarding temporal distribution, AEs were reported within one day post-vaccination in 70.31% of cases, whereas only 1.81% of reports occurred beyond 30 days after vaccination. The median time to onset of AEs was within 0 days after vaccination (**Figure 1B**). Weibull distribution analysis indicated an early failure pattern, characterized by acute reactions with short latency periods (**Table 2**).

**Table 1:** Characteristics of Tdap-related reports from VAERS.

Characteristics	Number of events (%)
Total	20,128 (100%)
Gender	
Male	7,240 (35.97%)
Female	12,199 (60.61%)
Unknown	689 (3.42%)
Age(year)	
< 10	1,058 (5.26%)
10–18	6,391 (31.75%)
19–64	9,594 (47.66%)
≥ 65	1,950 (9.69%)
Unknown	1,135 (5.64%)
Alone	
Yes	9,331 (46.36%)
No	10,797 (53.64%)
Serious	
Yes	846 (4.20%)
No	19,282 (95.80%)
Clinical outcome	
Died	29(0.14%)
Disability	313(1.56%)
Hospitalized	532(2.64%)
Life threatening	172(0.85%)
Prolonged hospitalization	17(0.08%)
Recovered	8,064(40.06%)
Unknown	11,001(54.66%)
Onset time (day)	
0–1	14,151 (70.31%)
1–30	3,332 (16.55%)
30+	364 (1.81%)
Unknown	2,281 (11.33%)

Tdap, tetanus, diphtheria, and acellular pertussis; VAERS, vaccine adverse event reporting system.

**Table 2:** Weibull distribution of Tdap-related AEs.

	Time to onset (d)		Weibull distribution		
Case, n	Median (IQR)	Min-max	Scale parameter (α 95% CI)	Shape parameter (β 95% CI)	Temporal pattern
17,847	0 (0–1)	0–32,872	0.227 (0.214-0.241)	0.259 (0.257-0.262)	Early failure

Tdap, tetanus, diphtheria, and acellular pertussis; AEs, adverse events; n, number of reports that include available time-to-onset data; IQR, interquartile range; CI, confidence interval; α, Weibull scale parameters; β, Weibull shape parameters. Temporal pattern classification was based on β: Early failure pattern (decreasing hazard over time) was defined as β < 1 with the upper bound of the 95%CI below 1.

### 3.2 Disproportionality analysis

In this study, 2,651 Tdap vaccine-associated PTs were identified spanning 27 SOCs. Using four complementary algorithms (ROR, PRR, BCPNN, MGPS), we identified 102 positive PT signals across 17 SOCs. The cross-method concordance suggested high reliability for the core group of shared signals; however, these findings do not establish causality. All signals remained statistically significant after FDR adjustment (**Supplementary Table 3**). Injection site erythema, injection site swelling, and erythema were the most frequently reported positive signals (**Table 3**). Syncope also showed high reporting frequencies and strong signal intensities, reflecting vasovagal responses associated with vaccination.

**Table 3:** Top 10 most frequently reported positive PT signals.

PT	p-adjust	N	ROR (95% CI)	PRR (X^2^)	EBGM (EBGM05)	IC (IC025)
Injection Site Erythema	< 0.001	2,658	2.77 (2.65-2.88)	2.53 (2495.93)	2.47 (2.37)	1.30 (1.24)
Injection Site Swelling	< 0.001	2,154	2.75 (2.63-2.88)	2.56 (2050.95)	2.50 (2.38)	1.32 (1.25)
Erythema	< 0.001	1,724	2.34 (2.23-2.46)	2.23 (1170.08)	2.18 (2.08)	1.12 (1.05)
Injection Site Warmth	< 0.001	1,219	2.65 (2.49-2.81)	2.55 (1125.15)	2.48 (2.34)	1.31 (1.22)
Syncope	< 0.001	1,208	3.43 (3.23-3.64)	3.28 (1848.27)	3.16 (2.98)	1.66 (1.57)
Wrong Product Administered	< 0.001	1,092	3.17 (2.95-3.4)	3.08 (1112.99)	2.98 (2.77)	1.57 (1.47)
Loss Of Consciousness	< 0.001	822	2.70 (2.52-2.90)	2.63 (796.34)	2.56 (2.39)	1.35 (1.25)
Skin Warm	< 0.001	808	5.26 (4.89-5.67)	5.10 (2404.09)	4.77 (4.43)	2.25 (2.14)
Pallor	< 0.001	787	2.75 (2.63-2.88)	2.56 (2050.95)	2.50 (2.38)	1.32 (1.25)
Exposure During Pregnancy	< 0.001	673	7.69 (7.09-8.35)	7.47 (3360.49)	6.74 (6.21)	2.74 (2.62)

Tdap, tetanus, diphtheria, and acellular pertussis; SOC, system organ class; PT, preferred term; ROR, reporting odds ratio; PRR, proportional reporting ratio; EBGM,empirical Bayesian geometric mean; IC, information component. Statistical significance is defined as p-adjust < 0.05.

We further conducted subgroup analyses to reduce demographic confounding and provide a multidimensional assessment of Tdap safety. Among positive signals that met the predefined criteria of all four algorithms and exceeded three reports, those further demonstrating robust disproportionality, potential clinical relevance, and clear subgroup specificity were prioritized for detailed evaluation. **Supplementary Table 4** summarized the priority signals identified in distinct subgroups, with their respective clinical significance ratings.

#### 3.2.1 PTs across age subgroups

**Figure 2:**
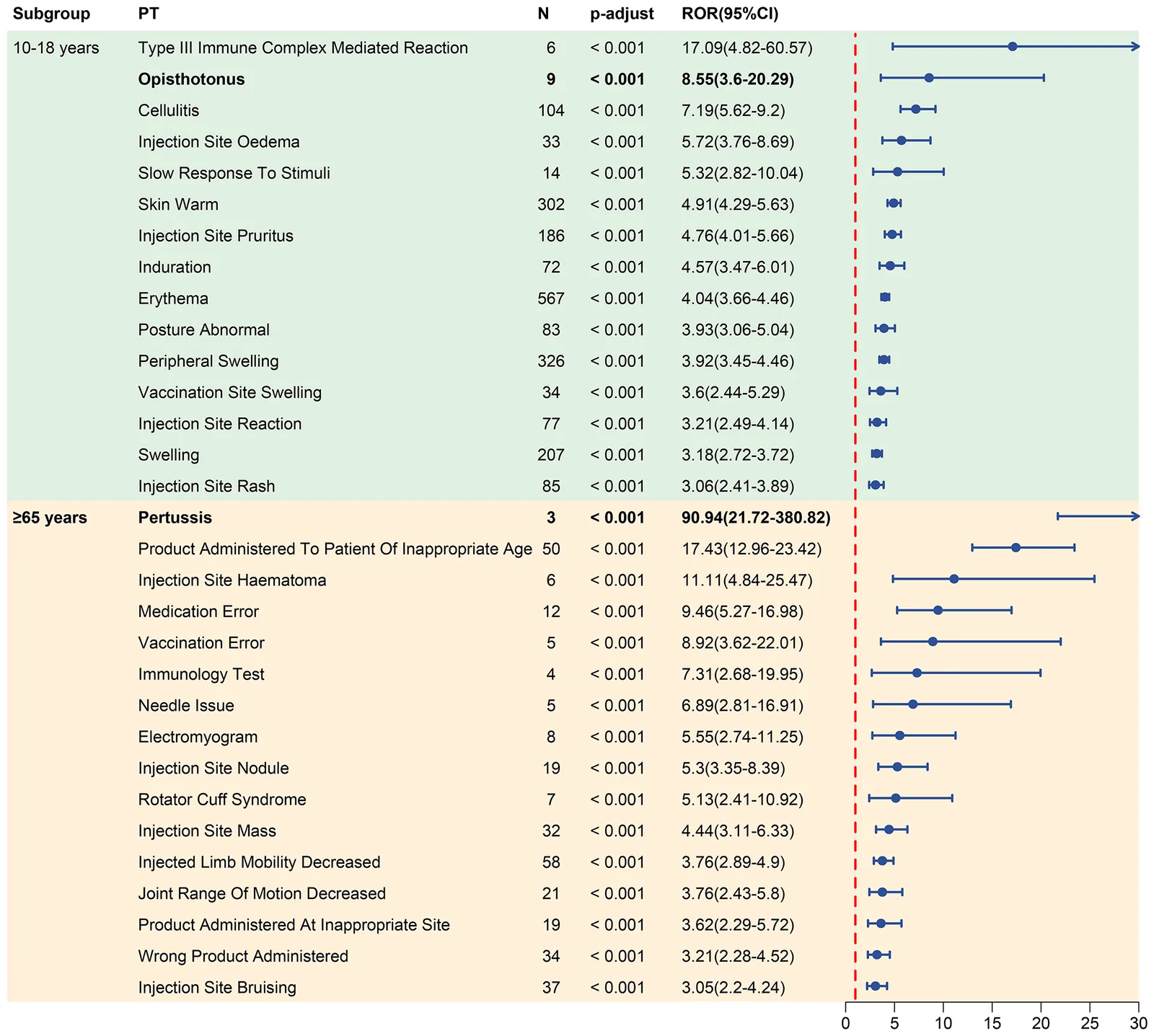
Forest plot of age-specific positive PT signals for Tdap vaccination. PT, preferred term; Tdap, tetanus, diphtheria, and acellular pertussis. Statistical significance is defined as p-adjust < 0.05. Bold text indicates signals showing robust disproportionality, potential clinical relevance, and clear subgroup specificity. The red dashed line indicates the null value (ROR = 1). Data points to the right of the line represent a higher reporting frequency compared to other vaccines.

In the age-based subgroup analyses, we focused on adolescents aged 10 to 18 years and older adults aged ≥65 years. A total of 23 and 24 positive signals were identified in the adolescents and ≥65 age subgroups, respectively (**Supplementary Tables 5**, **S6**). Injection site reactions predominated in both subgroups. The ≥65 age subgroup yielded 16 specific signals, with pertussis showing the strongest disproportionality (**Figure 2**).

Among the 15 signals unique to the adolescents, opisthotonus showed strong disproportionality (N = 9, ROR = 8.55) (**Figure 2**). Although the lower confidence bound of the signal was well above the pre-specified threshold, the wide confidence interval and small number of reports indicated limited precision of this signal, warranting cautious interpretation. Among the nine reports of opisthotonus, eight described acute, self-limiting events, whereas one characterized the event as a seizure-related manifestation. Of these nine cases, five were male and four female, with symptom onset occurring on the day of vaccination in eight cases. Frequently co-reported symptoms included eye movement disorder, loss of consciousness and pallor (**Supplementary Table 7**).

#### 3.2.2 PTs across sex subgroups

**Figure 3:**
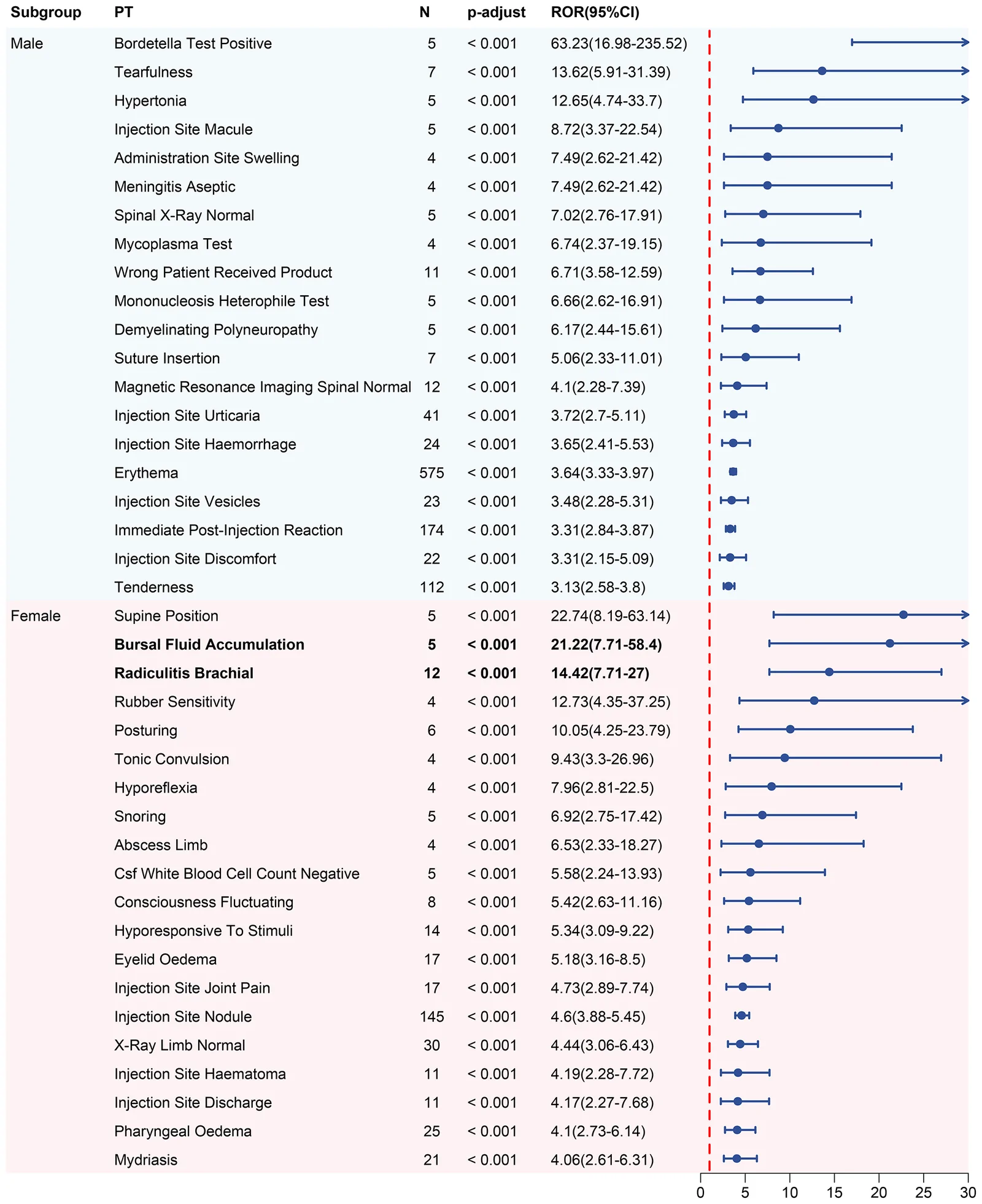
Forest plot of sex-specific positive PT signals for Tdap vaccination. PT, preferred term; Tdap, tetanus, diphtheria, and acellular pertussis. Statistical significance is defined as p-adjust < 0.05. Bold text indicates signals showing robust disproportionality, potential clinical relevance, and clear subgroup specificity. The red dashed line indicates the null value (ROR = 1). Data points to the right of the line represent a higher reporting frequency compared to other vaccines.

Sex-stratified disproportionality analysis identified 57 positive signals in the male subgroup and 67 in the non-pregnant female subgroup (**Supplementary Tables 8**, **S9**). Both groups showed a similar pattern of commonly reported adverse events, with injection site erythema and injection site swelling among the most frequently reported positive signals. There was substantial signal overlap between the two groups, but 24 signals were unique to males and 34 were exclusive to the non-pregnant female subgroup (**Figure 3**). In the male subgroup, hypertonia was the signal with a notably elevated reporting odds ratio.

Among the female-specific signals, bursal fluid accumulation (N = 5, ROR = 21.22) and radiculitis brachial (N = 12, ROR = 14.42) exhibited significant disproportionality. However, given the small number of reports and the wide confidence intervals, these findings should be interpreted with caution. Furthermore, we analyzed the clinical characteristics and potential severity of the two positive signals identified in the non-pregnant female subgroup. The 12 female cases of radiculitis brachial predominantly involved middle-aged and older adults (aged 36–75 years). Three cases were classified as serious due to disability. Commonly reported concomitant symptoms included pain in extremity and muscular weakness. All 12 cases received Tdap alone, with no concomitant vaccinations reported. Notably, of the 12 reported cases, 10 were consistent with the typical clinical presentation; however, only 6 received a formal physician diagnosis and merely 3 were radiologically confirmed. The median time to AE onset was 1 day (range: 0–15 days), with 7 reports describing pain onset on the day of vaccination or within 24 hours. A detailed case-by-case clinical review of these 12 cases, encompassing time-to-onset, clinical features, diagnostic workup, and final outcomes, is provided in **Supplementary Table 7**. Among the five female reports of bursal fluid accumulation, all occurred in patients aged 37 to 52 years with symptom onset within 24 hours of vaccination. One case was classified as serious. Four of the five patients received Tdap alone, with only one case involving co-administration. Additional clinical details on radiculitis brachial and bursal fluid accumulation were provided in **Supplementary Table 8**.

#### 3.2.3 PTs across pregnant individuals

To better understand the safety of Tdap in pregnant individuals, we identified and analyzed 744 pregnancy-related reports (**Table 4**). In 647 (86.96%) reports, the Tdap vaccine was given alone. The median maternal age at vaccination was 30 years (range 14–47 years). Among the 549 reports (73.79%) with known gestational age, the majority of immunizations (N = 462) occurred during the third trimester. Two hundred and twelve(28.49%) reports did not describe any AE. A total of 73 (9.81%) reports were classified as serious, and no maternal mortality reports were identified.

**Table 4:** Characteristics of VAERS reports following Tdap administration in pregnant women (n = 744).

Characteristics	
Total reports	
Reports with no adverse events, N(%)	212 (28.49%)
Maternal age in years, median (range)	30 (14–47)
Interval from vaccination to adverse event in days, median (range)	0 (0–1084)
Reports of serious adverse events, N(%)	73 (9.81%)
Maternal age groups, N(%)	
10–17 years	14 (1.88%)
18–29 years	300 (40.32%)
30–39 years	340 (45.70%)
≥ 40 years	26 (3.49%)
Unknown	64 (8.60%)
Vaccines administered,N(%)	
TDAP alone	647 (86.96%)
Other combinations	97 (13.04%)
Trimester of pregnancy at time of vaccination (N = 549), N(%)	
First (0–13 weeks)	34 (4.57%)
Second (14–27 weeks)	53 (7.12%)
Third (28 + weeks)	462 (62.10%)

VAERS, vaccine adverse event reporting system; Tdap, tetanus, diphtheria, and acellular pertussis.

Adverse events reported in pregnancy often involve multiple medical conditions, which can be classified as non-pregnancy-specific, pregnancy-specific, and fetus-related conditions. Among non-pregnancy-specific conditions, general disorders and administration site conditions were most common (**Table 5**). In addition, 45 reports were submitted due to the administration of additional vaccine doses, and none of these documented any adverse reactions. The most frequent pregnancy-specific condition was premature delivery (N = 16), followed by premature labour (N = 12) and stillbirth (N = 10). Two reports described amniotic cavity infection. Fetal outcomes commonly reported included fetal death (N = 10) and fetal hypokinesia (N = 10). One case of birth defects were reported. The infant was born with a MTHFR heterozygous gene mutation of C677T type and was diagnosed with severe ankyloglossia and severe labial frenulum restriction.

**Table 5:** Primary PTs in pregnant women and infants following maternal Tdap administration.

PT	N (‰)
Pregnancy-specific outcomes	
Premature Delivery	16 (5.59‰)
Premature Labour	12 (4.20‰)
Stillbirth	10 (3.50‰)
Premature Rupture Of Membranes	9 (3.15‰)
Pre-Eclampsia	5 (1.75‰)
Premature Separation Of Placenta	5 (1.75‰)
Gestational Diabetes	4 (1.40‰)
Induced Labour	4 (1.40‰)
Preterm Premature Rupture Of Membranes	2 (0.70‰)
Gestational Hypertension	2 (0.7‰)
Placenta Praevia	2 (0.70‰)
Placental Disorder	2 (0.70‰)
Haemorrhage In Pregnancy	2 (0.70‰)
Amniotic Cavity Infection	2 (0.70‰)
Vaginal Haemorrhage	2 (0.70‰)
Abortion Spontaneous	1 (0.35‰)
Nonpregnancy-specific outcomes (top 10)	
Pain	70 (24.48‰)
Injection Site Pain	61 (21.33‰)
Pain In Extremity	57 (19.93‰)
Pyrexia	49 (17.13‰)
Product Storage Error	47 (16.43‰)
Injection Site Swelling	46 (16.08‰)
Extra Dose Administered	45 (15.73‰)
Injection Site Erythema	44 (15.38‰)
Nausea	43 (15.03‰)
Dizziness	33 (11.53‰)
Infant/neonatal outcomes	
Foetal Hypokinesia	10 (3.50‰)
Foetal Death	10 (3.50‰)
Foetal Disorder	8 (2.80‰)
Foetal Heart Rate Abnormal	7 (2.45‰)
Neonatal Disorder	6 (2.10‰)
Foetal Growth Restriction	2 (0.70‰)

PT, preferred term; Tdap, tetanus, diphtheria, and acellular pertussis.

**Figure 4:**
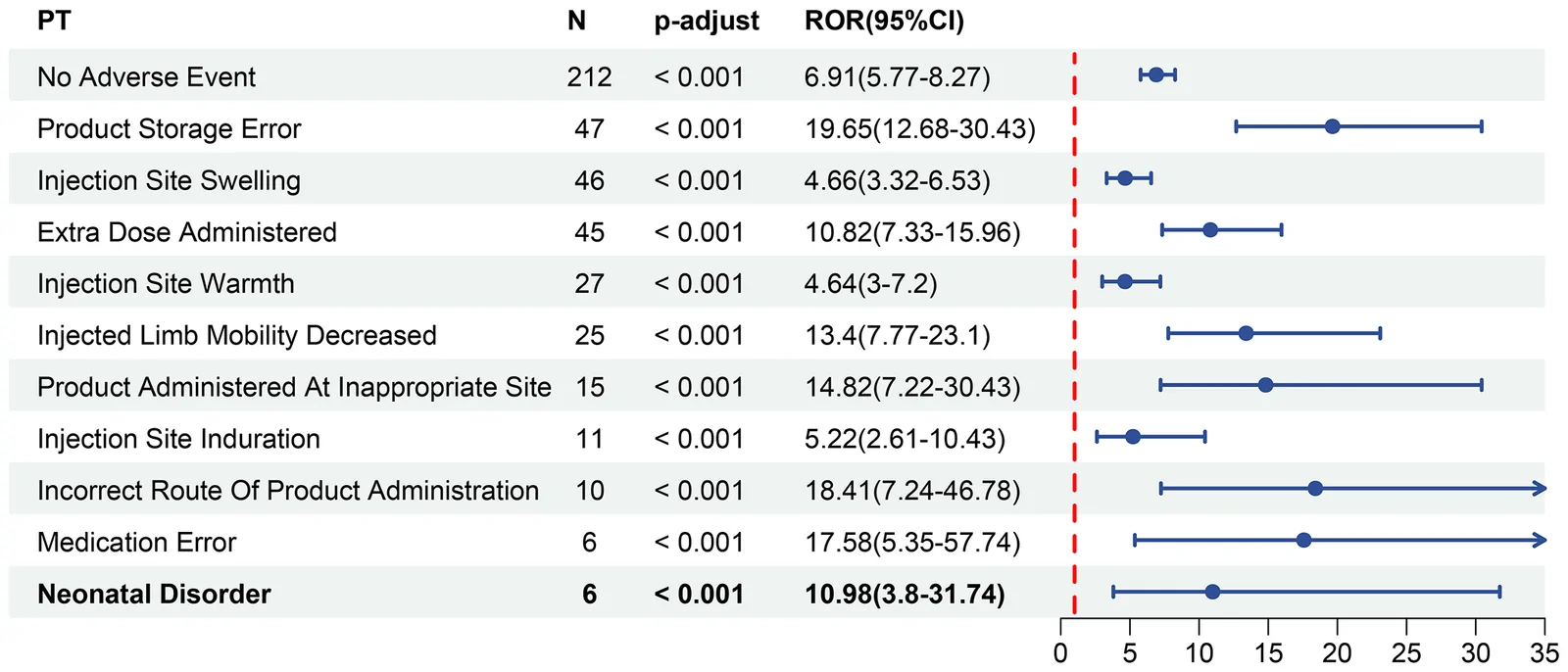
Forest plot of positive PT signals for Tdap vaccination in pregnant women. PT, preferred term; Tdap, tetanus, diphtheria, and acellular pertussis. Statistical significance is defined as p-adjust < 0.05. Bold text indicates pregnancy-specific or fetal-related PTs. The red dashed line indicates the null value (ROR = 1). Data points to the right of the line represent a higher reporting frequency compared to other vaccines.

Disproportionality analysis indicated elevated reporting odds for neonatal disorders (**Figure 4**; **Supplementary Table 11**). Six reports contained the code ‘Neonatal disorder’, including fetal hypokinesia (N = 1), birth defects (N = 1), infant hearing concerns with hospital confirmation of normal hearing and vision (N = 1), neonatal jaundice (N = 1), gastrointestinal issues (N = 1), and perinatal ischemic stroke of the left middle cerebral artery (N = 1). No other positive signals for pregnancy-specific and fetus-related PTs were identified.

### 3.3 Sensitivity analysis

We conducted a complete-case sensitivity analysis in the non-pregnant female subgroup. After exclusion of reports with unknown key variables, the main disproportionality signals remained generally unchanged in direction and magnitude. supporting the robustness of the observed associations (**Supplementary Table 12**). No materially new major signals emerged, and the overall pattern of the principal signals remained stable. In the single-vaccine Tdap sensitivity analysis, the signals for radiculitis brachial and bursal fluid accumulation in non-pregnant females remained robust (**Supplementary Table 13**). Conversely, the opisthotonus signal in the adolescent subgroup was no longer statistically significant, suggesting the original signal may have been confounded by co-administration. Of the 9 adolescent opisthotonus reports from the primary analysis, 7 involved concomitant vaccination, with 6 specifically including MenACWY. In the case-deletion analysis, the signal for radiculitis brachial in non-pregnant females remained statistically significant after removing up to 7 cases but became non-significant after removing the 8th case (**Supplementary Table 14**). In addition, we performed a robustness analysis restricting radiculitis brachial cases to those with onset within 28 days post-vaccination (**Supplementary Table 15**). It revealed a robust and slightly strengthened signal for radiculitis brachial (N = 11, ROR = 15.23). This temporal clustering strengthens the evidence for a temporal association between Tdap vaccination and radiculitis brachial in non-pregnant females.

## 4 Discussion

### 4.1 Overall safety profile in the total population

This study conducted a comprehensive analysis of Tdap-related adverse reaction reports from the VAERS database over the past decade. Overall, Tdap demonstrated a favorable safety profile, with the vast majority of reported adverse events being acute and non-serious. The most frequently reported positive safety signals were injection site erythema, injection site swelling, and erythema. Syncope also emerged as a commonly reported positive signal. Syncope typically occurred within 15 minutes of vaccination and is generally classified as an immediate vasovagal reaction rather than a true vaccine-related neurological disorder (Centers for Disease Control and Prevention (CDC), 2008). From a clinical perspective, syncope remains an important safety concern due to the secondary risk of falls and head injuries. Therefore, 15-minute post-vaccination observation is a practical and important measure to reduce preventable harm. The adverse event profile, characterized by local reactions and mild systemic symptoms, aligns closely with prior data and reinforces the overall good tolerability of Tdap in the general population.

### 4.2 Age-specific safety signals

In the adolescent subgroup, our study identified a positive signal for opisthotonus. However, the sensitivity analysis restricted to Tdap administered alone showed that this signal was no longer positive. This finding suggests that concomitant vaccination may have substantially contributed to the observed signal in the primary analysis. Consistent with this interpretation, case review indicated that 7 of the 9 adolescent opisthotonus reports involved co-administration, including 6 reports with MenACWY. Therefore, attribution of this signal specifically to Tdap should be made with caution. Moreover, further case review revealed that the vast majority of events contributing to this signal (8 out of 9) were characterized by acute onset and rapid resolution. This suggests that these events likely represent transient, non-specific acute dystonia or functional neurological symptoms rather than organic pathology.

Among adults aged ≥ 65 years, adverse events were predominantly local reactions, consistent with prior VAERS safety assessments of Tdap in this age group. We also observed a strong signal for pertussis in this age group. However, this signal is unlikely to represent a clinically meaningful vaccine adverse effect. A more plausible explanation attributes this phenomenon to altered reporting behaviors triggered by coincidental medical events following vaccination. In clinical practice, breakthrough infections or vaccine failure cases following vaccination are more likely to be reported to VAERS because they are clinically unexpected. Additionally, it is highly plausible that some of these reports actually reflect coincidental natural infections rather than vaccine-related events, a distinction not documented in the VAERS narratives. For example, individuals could have been vaccinated during the pertussis incubation period or shortly after symptom onset, with pertussis being diagnosed subsequently. Thus, this signal likely reflects the complexities of disease reporting in passive surveillance rather than a direct safety concern attributable to the vaccine itself.

### 4.3 Signals specific to non-pregnant females

While the sex-stratified analysis excluding pregnancy showed largely overlapping safety patterns, some differences were observed. Specifically, radiculitis brachial and bursal fluid accumulation were identified as positive signals only in the non-pregnant female subgroup. Sensitivity analyses yielded consistent findings. Nevertheless, signals derived from a limited number of reports are statistically unstable and have inherently low positive predictive value; thus, they should be interpreted with extreme caution and prioritized for validation in larger datasets. Additionally, for radiculitis brachial, the observed early-onset pattern in our dataset diverges from the classical 7–10 day window described in the literature, suggesting that these reports may represent a heterogeneous mix of true neuritis and other acute peri-vaccinal events. Most reports also lacked electrodiagnostic or imaging confirmation. Consequently, diagnostic misclassification, protopathic bias, and reporting biases cannot be excluded. Given these limitations, this signal should not be regarded as evidence of a confirmed biologically mediated association in the absence of external validation.

The MedDRA preferred term for “radiculitis brachial” is clinically regarded as the more widely recognized condition “brachial neuritis” or “Parsonage-Turner syndrome.” Brachial neuritis is a rare and distinct immune-mediated peripheral neuropathy characterized by severe shoulder and arm pain, followed by multifocal paresis and atrophy of the upper extremity muscles (Mizrahi and Spinner, 2017). Brachial neuritis is traditionally regarded as a self-limiting condition, with most patients achieving recovery within two or three years. However, more recent evidence indicates that 50% to 67% of individuals sustain prolonged pain and paresis, which significantly impairs their daily life and employment (Van Alfen, 2011). Moreover, effective treatments for this condition remain limited. Early pain is managed with oral corticosteroids and analgesics, while the subsequent recovery phase focuses on rehabilitation. Vaccination is one of the known potential triggers of brachial neuritis. Several vaccines have been reported to be temporally associated with brachial neuritis, including influenza vaccines, human papillomavirus vaccines, COVID-19 vaccines, and tetanus-containing vaccines (Debeer et al., 2008; Loganathan et al., 2023; Shaikh et al., 2012). A report by the Institute of Medicine (IOM) found that evidence favored a causal relationship between receipt of tetanus toxoid and brachial neuritis, although the absolute risk is extremely low (Stratton, 1994). The occurrence of brachial neuritis across various vaccines suggests that it is not a toxic reaction to a specific vaccine adjuvant or antigenic component, but rather is associated with non-specific immune activation following vaccination. Although the pathogenesis of vaccine-associated brachial neuritis remains elusive, it is thought that genetic predisposition, biomechanical factors (repetitive or strenuous motor tasks), and autoimmune response contribute to its development (Van Eijk et al., 2016).

Although brachial neuritis classically exhibits male predominance in the general population, the female-specific signal observed here warrants careful interpretation (Van Alfen et al., 2015; Van Eijk et al., 2016). From a biological perspective, gender influences the immune response to vaccination. Current evidence suggests that females generally exhibit stronger innate and adaptive immune responses than males (Klein and Flanagan, 2016). This divergence is heavily influenced by sex hormones. Estrogens potentiate dendritic cell function and B-cell activation, while androgens exert immunosuppressive effects (Chakraborty et al., 2023). Although the above theory offers a plausible explanation for the signal observed exclusively in non-pregnant females, the underlying mechanism of vaccine-associated brachial neuritis remains unproven. Beyond biological hypotheses, differential reporting behaviors likely contribute to this disparity. Females are significantly more inclined to seek medical attention for new symptoms (Ballering et al., 2023). In VAERS, this behavioral differences introduce artificially inflating the apparent reporting frequency of brachial neuritis among females independent of true incidence. Furthermore, this signal is likely subject to confounding by variations in vaccinee demographics and multiple comparisons. Accordingly, it should be interpreted strictly as an exploratory finding for hypothesis generation rather than evidence of causality. Future validation requires studies leveraging active surveillance platforms with adjudicated case definitions.

Bursal fluid accumulation is clinically compatible with Shoulder Injury Related to Vaccine Administration (SIRVA), a recognized complication of improper intramuscular injection technique (Jenkins and Duckworth, 2023). Rather than indicating a novel safety problem, this signal likely reflects a clinically recognizable injection-related phenotype. Its emergence as a positive signal in females carries biological plausibility. Compared with males, females generally have smaller deltoid muscle mass and shorter humeral length. It has been hypothesized that if needle length and injection site are not appropriately adjusted for individual anthropometric characteristics, vaccine components may inadvertently be deposited into the subdeltoid bursa or other pericapsular tissue, triggering an inflammatory response (Atanasoff et al., 2010; Kutaish et al., 2025). While this anatomical hypothesis is compelling, the signal remains influenced by other sex-related differences such as immune responses and reporting behavior. Therefore, these explanations should be considered hypotheses rather than established mechanisms. The signal for bursal fluid accumulation underscores the importance of personalized assessment and proper intramuscular injection technique, particularly in women with smaller deltoid masses.

### 4.4 Pregnancy-specific safety signals

In the pregnant population, we did not find any new or unexpected patterns of maternal or fetal adverse events. Although broad MedDRA terms such as “Neonatal disorders” showed disproportionate reporting, clinical review did not identify any unexpected clusters of adverse events. This suggests that these signals likely stem from non-specific coding practices rather than reflecting genuine safety concerns. It is crucial to note that these findings should be interpreted cautiously. The number of pregnancy-related reports in VAERS limits the statistical power to detect very rare events, and spontaneous-report systems cannot establish the absence of risk. Therefore, our results should not be interpreted as independently proving safety in pregnancy; rather, these indicate that no unexpected disproportionality signals were detected in this dataset. Our findings are broadly consistent with high-quality evidence on the safety of Tdap vaccination during pregnancy. Multiple cohort studies, Vaccine Safety Datalink analyses, and systematic reviews have demonstrated that administering Tdap during pregnancy is not associated with an increased risk of adverse pregnancy or neonatal outcomes (Greenberg et al., 2023; Panagiotakopoulos et al., 2020; Haidara et al., 2024). Notably, we observed no elevated signal for chorioamnionitis in the pregnant subgroup. While some earlier studies suggested a possible modest increase in chorioamnionitis risk following Tdap vaccination during pregnancy, the evolving evidence provides critical context for interpreting these findings (Kharbanda et al., 2014; Vygen-Bonnet et al., 2020). Recent large-scale VSD cohort studies have demonstrated that the observed association was driven by immortal time bias and non-specific diagnostic coding, rather than by a true biological risk. Chart reviews in the VSD study confirmed no increased risk of true chorioamnionitis or adverse infant outcomes following Tdap vaccination during pregnancy (Greenberg et al., 2023). Overall, our findings yield no unexpected signals and are generally consistent with the current recommendations for routine maternal immunization.

### 4.5 Limitations

This study has several limitations. First, VAERS lacks denominator data and is limited by reporting biases, such as underreporting, stimulated reporting, and incomplete reporting. As a result, incidence rates cannot be calculated, and direct risk comparisons across subgroups are not feasible (Varricchio et al., 2004; Shimabukuro et al., 2015). Second, although we conducted subgroup analyses and detailed clinical reviews, residual confounding cannot be fully ruled out. Reporting biases and coding variations persist. For example, AEs in adolescents and pregnant individuals are more likely to be reported due to heightened awareness, and certain PTs are more commonly observed in specific subgroups. Notably, subgroup-level associations should not be extrapolated to individual patients. Third, important clinical information is often missing in VAERS reports. Missing underlying conditions and concomitant medications may lead to confounding by indication and protopathic bias. The healthy vaccinee effect also may lead to bias. The absence of laboratory confirmation and the reliance on reporter-assigned diagnoses may cause outcome misclassification. Missing essential data like age, sex, and time of onset can bias subgroup and time-to-onset analyses. Fourth, we did not evaluate the contribution of repeat vaccinations or specific co-administered vaccines to the identified signals. These issues warrant further investigation in future studies. Fifth, multiple statistical comparisons were performed; consequently, some signals may represent false-positive findings despite FDR adjustment and the use of multiple signal detection methods. Although FDR correction was applied within each subgroup, it does not control the overall Type I error rate across all subgroup comparisons combined, thereby elevating the risk of false positives, particularly for rare events with small case counts. Consequently, all subgroup-specific signals must be interpreted strictly as exploratory hypotheses requiring external validation rather than definitive clinical findings. In addition, VAERS is not powered to detect very rare adverse events, and standard signal thresholds may miss uncommon but clinically important outcomes. Therefore, the absence of unexpected signals, particularly in pregnant or older adult subgroups, should not be interpreted as evidence of safety. Finally, due to regional variations in reporting patterns and the lack of detailed population diversity data, VAERS data may not be fully representative of the overall U.S. population, and the generalizability to other countries and specific racial groups is limited. Our findings are contextualized within current immunization policies, and the introduction of new pertussis vaccines or changes in vaccination recommendations could affect the safety profile. Applied as an interpretive lens, the Bradford Hill framework indicates that our data provide only limited support for temporality (given the post-vaccination onset) and indirect support for plausibility and coherence (informed by external literature on vaccine-related mechanisms). However, the limitations of this study preclude adequate assessment of the remaining criteria, including strength, specificity, biological gradient, experiment, and analogy. Accordingly, the detected signals indicate statistical associations only and do not imply causality. These findings should be interpreted with caution and regarded primarily as hypothesis-generating rather than confirmatory.

### 4.6 Clinical and public health implications

#### 4.6.1 Clinical implications

Our findings generally support the established safety profile of Tdap vaccination while highlighting several subgroup-specific signals that may merit further attention. For non-pregnant females, although the absolute risk is likely very low, clinicians should be aware of the potential for brachial neuritis following Tdap vaccination. Delayed recognition of this condition may lead to prolonged symptoms or disability. The signals for bursal fluid accumulation underscore the importance of proper injection technique, including correct landmarking, appropriate needle length selection, and avoidance of excessively high deltoid administration. For pregnant individuals and older adults, the absence of unexpected safety signals in this analysis supports continued adherence to current recommendations for Tdap administration, while underscoring the need for ongoing surveillance.

For vaccine recipients, it is essential to recognize that the benefits of Tdap vaccination greatly outweigh the risks and to strictly adhere to the recommended post-vaccination observation period of at least 15 minutes. However, individuals should seek medical advice if they experience specific warning signs, such as: intense arm pain followed by weakness or numbness, severe shoulder pain with limited arm movement, unusual muscle spasms, or other symptoms that are severe, unusual, or do not improve. Pregnant vaccine recipients should continue routine obstetric follow-up and seek assessment for any concerning maternal symptoms.

#### 4.6.2 Public health implications

At the public health level, this study supports current Tdap vaccination recommendations, which are important for maintaining public confidence. Furthermore, the identification of subgroup-specific signals demonstrates the value of VAERS as a signal detection system and underscores the importance of implementing stratified pharmacovigilance. VAERS findings are hypothesis-generating rather than confirmatory. Therefore, the findings of this study should not directly alter current vaccination policies. Instead, their utility lies in identifying the most plausible safety signals that warrant risk quantification through rigorous epidemiological validation. Specifically, future research should utilize the self-controlled case series (SCCS) design within active surveillance systems, or conduct retrospective cohort studies with well-defined denominators to calculate absolute risks. If validated, the potential subgroup-specific safety signals could inform more nuanced risk-benefit assessments and targeted monitoring strategies. Importantly, targeted clinician education should be implemented now to improve the recognition and reporting of uncommon neurologic and injection-related events. Moreover, risk communication should place rare adverse events in the context of the substantial benefits of Tdap vaccination to preserve public confidence without amplifying hesitancy.

## 5 Conclusion

VAERS surveillance data from the past decade reaffirm that Tdap vaccination has a favorable safety profile in the general population, as well as in older adults and pregnant individuals, with most reported adverse events being non-serious and self-limiting. The female-specific signals of bursal fluid accumulation and radiculitis brachial, if confirmed, highlights the potential for subgroup-specific patterns in vaccine-related adverse events. Given the inherent limitations of passive surveillance systems, these findings cannot be used to infer causality. Further validation through active surveillance systems or large-scale epidemiological studies is warranted. Overall, our results support the safety of current Tdap vaccination strategies and underscore the critical role of ongoing pharmacovigilance in ensuring vaccine safety.

## Data Availability

The original contributions presented in the study are included in the article/**Supplementary Material**. Further inquiries can be directed to the corresponding author.
