# Supplementary material for: Post-marketing safety surveillance of tetanus, diphtheria, and acellular pertussis (Tdap) vaccine: a disproportionality analysis of the vaccine adverse event reporting system (2015–2025)
Source: Front Cell Infect Microbiol. 2026 Sep 8;16:1831548. doi: 10.3389/fcimb.2026.1831548 (PMC13593522; doi:10.3389/fcimb.2026.1831548)
Supplement: Supplementary file 1 [file Table1.docx]

**Supplementary Material**

[Table S1. 2 × 2 contingency table for disproportionality analysis.	2](#_Toc19901)

[Table S2. Four major algorithms used for signal detection.	3](#_Toc25151)

[Table S3. Signal strength of all Tdap vaccine-related positive PT signals and their corresponding SOCs.	4](#_Toc14006)

[Table S4. Clinical significance rating of subgroup-specific priority signals.	9](#_Toc10826)

[Table S5. Signal strength of Tdap vaccine-related positive PT signals and their corresponding SOCs in adolescents aged 10–18 years.	10](#_Toc18387)

[Table S6. Signal strength of Tdap vaccine-related positive PT signals and their corresponding SOCs in older adults aged ≥65 years.	12](#_Toc25819)

[Table S7. Clinical case-by-case review for radiculitis brachial signal in non-pregnant female subgroup.	14](#_Toc20877)

[Table S8. Clinical details of subgroup-specific priority signals.	17](#_Toc4612)

[Table S9. Signal strength of Tdap vaccine-related positive PT signals and their corresponding SOCs in male.	18](#_Toc1832)

[Table S10. Signal strength of Tdap vaccine-related positive PT signals and their corresponding SOCs in non-pregnant females.	21](#_Toc26001)

[Table S11. Signal strength of Tdap vaccine-related positive PT signals and their corresponding SOCs in pregnant females.	24](#_Toc11403)

[Table S12. Signal strength of Tdap vaccine-related positive PT signals and their corresponding SOCs in complete-case sensitivity analysis among non-pregnant females.	25](#_Toc7660)

[Table S13. Single-vaccine Tdap sensitivity analysis for the signal of radiculitis brachial and bursal fluid accumulation in non-pregnant females, opisthotonus in the adolescents .	28](#_Toc28691)

[Table S14. Case-deletion analysis for the signal of radiculitis brachial following Tdap vaccination in non-pregnant females.	29](#_Toc23342)

[Table S15. Restricted time-window disproportionality analysis for the signal of radiculitis brachial following Tdap vaccination in non-pregnant females.	30](#_Toc14299)

# Table S1. 2 × 2 contingency table for disproportionality analysis.

|  | No. target adverse reaction reports | No. other adverse reaction reports | Total |
| --- | --- | --- | --- |
| Target drug | a | b | a + b |
| Other drugs | c | d | c + d |
| Total | a + c | b + d | N = a + b + c + d |

a, number of reports containing both the suspect drug and the suspect adverse drug reaction; b, number of reports containing the suspect adverse drug reaction with other medications (except the drug of interest); c, number of reports containing the suspect drug with other adverse drug reactions (except the event of interest); d, number of reports containing other medications and other adverse drug reactions.

# Table S2. Four major algorithms used for signal detection.

| Method | Formula | Threshold |
| --- | --- | --- |
| ROR | $ROR=\frac{a/c}{b/d}=\frac{ad}{bc}$  $95\% CI=e^{ln\left( ROR \right)\pm1.96\sqrt{(\frac{1}{a}+\frac{1}{b}+\frac{1}{c}+\frac{1}{d})}}$ | a ≥ 3 and lower limit of 95% CI > 1 |
| PRR | $PRR=\frac{a/{(a+b)}}{c/{(c+d)}}$  $\chi^{2}=\frac{{(a+b+c+d)[(ad-bc)}^{2}]}{(a+b)(c+d)(b+d)(a+c)}$ | a ≥ 3 and PRR ≥ 2 and χ^2^ ≥ 4 |
| BCPNN | $IC={log}_{2}^{\frac{p(x,y)}{p\left( x \right)p(y)}}={log}_{2}^{\frac{a(a+b+c+d)}{(a+b)(a+c)}}$  *95%IC*$=E\left( IC \right)-2\sqrt{V(IC)}$ | IC025 > 0 |
| MGPS | $EBGM=\frac{a(a+b+c+d)}{(a+c)(a+b)}$  $EBGM05=e^{\ln\left( EBGM \right)-1.96\sqrt{(\frac{1}{a}+\frac{1}{b}+\frac{1}{c}+\frac{1}{d})}}$ | EBGM05 > 2 |

ROR, reporting odds ratio; CI, confidence interval; a, the number of reports; PRR, proportional reporting ratio; χ2, chi-squared; BCPNN, Bayesian confidence propagation neural network; IC, information components; IC025, the lower limit of 95% CI of the IC; E(IC), the IC expectations; V(IC), the variance of IC; MGPS, multi-item gamma Poisson shrinker; EBGM05, the lower 95% one-side CI of EBGM.

# Table S3. Signal strength of all Tdap vaccine-related positive PT signals and their corresponding SOCs.

| SOC | PT | a | p–adjust | ROR(95%CI) | PRR(X²) | EBGM(EBGM05) | IC(IC025) |
| --- | --- | --- | --- | --- | --- | --- | --- |
| General disorders and administration site conditions | Injection site erythema | 2658 | < 0.001 | 2.77(2.65–2.88) | 2.53(2495.93) | 2.47(2.37) | 1.30(1.24) |
| General disorders and administration site conditions | Injection site swelling | 2154 | < 0.001 | 2.75(2.63–2.88) | 2.56(2050.95) | 2.50(2.38) | 1.32(1.25) |
| Skin and subcutaneous tissue disorders | Erythema | 1724 | < 0.001 | 2.34(2.23–2.46) | 2.23(1170.08) | 2.18(2.08) | 1.12(1.05) |
| General disorders and administration site conditions | Injection site warmth | 1219 | < 0.001 | 2.65(2.49–2.81) | 2.55(1125.15) | 2.48(2.34) | 1.31(1.22) |
| Nervous system disorders | Syncope | 1208 | < 0.001 | 3.43(3.23–3.64) | 3.28(1848.27) | 3.16(2.98) | 1.66(1.57) |
| Injury, poisoning and procedural complications | Wrong product administered | 1092 | < 0.001 | 5.84(5.48–6.22) | 5.58(3784.34) | 5.18(4.86) | 2.37(2.27) |
| Nervous system disorders | Loss of consciousness | 822 | < 0.001 | 3.17(2.95–3.40) | 3.08(1112.99) | 2.98(2.77) | 1.57(1.47) |
| Skin and subcutaneous tissue disorders | Skin warm | 808 | < 0.001 | 2.70(2.52–2.90) | 2.63(796.34) | 2.56(2.39) | 1.35(1.25) |
| Vascular disorders | Pallor | 787 | < 0.001 | 5.26(4.89–5.67) | 5.10(2404.09) | 4.77(4.43) | 2.25(2.14) |
| Injury, poisoning and procedural complications | Exposure during pregnancy | 673 | < 0.001 | 7.69(7.09–8.35) | 7.47(3360.49) | 6.74(6.21) | 2.74(2.62) |
| General disorders and administration site conditions | Injection site induration | 497 | < 0.001 | 3.09(2.82–3.38) | 3.04(651.38) | 2.94(2.68) | 1.55(1.42) |
| General disorders and administration site conditions | Injected limb mobility decreased | 436 | < 0.001 | 3.66(3.32–4.03) | 3.60(775.53) | 3.45(3.13) | 1.78(1.64) |
| General disorders and administration site conditions | Immediate post–injection reaction | 432 | < 0.001 | 2.38(2.16–2.62) | 2.35(324.26) | 2.30(2.08) | 1.19(1.05) |
| General disorders and administration site conditions | Tenderness | 349 | < 0.001 | 2.36(2.12–2.63) | 2.34(259.53) | 2.29(2.05) | 1.19(1.03) |
| Infections and infestations | Cellulitis | 315 | < 0.001 | 3.86(3.44–4.33) | 3.81(616.17) | 3.64(3.25) | 1.85(1.68) |
| General disorders and administration site conditions | Injection site mass | 213 | < 0.001 | 2.52(2.20–2.90) | 2.51(185.71) | 2.44(2.13) | 1.28(1.08) |
| General disorders and administration site conditions | Induration | 207 | < 0.001 | 3.10(2.69–3.57) | 3.08(277.09) | 2.98(2.59) | 1.56(1.35) |
| Injury, poisoning and procedural complications | Head injury | 205 | < 0.001 | 3.80(3.30–4.38) | 3.78(394.15) | 3.61(3.13) | 1.83(1.63) |
| Musculoskeletal and connective tissue disorders | Musculoskeletal pain | 189 | < 0.001 | 2.89(2.50–3.35) | 2.87(220.84) | 2.79(2.41) | 1.46(1.25) |
| Nervous system disorders | Presyncope | 189 | < 0.001 | 2.67(2.30–3.09) | 2.65(186.73) | 2.58(2.23) | 1.35(1.14) |
| General disorders and administration site conditions | Injection site nodule | 177 | < 0.001 | 4.14(3.55–4.83) | 4.11(390.58) | 3.91(3.35) | 1.94(1.72) |
| Musculoskeletal and connective tissue disorders | Joint range of motion decreased | 171 | < 0.001 | 2.80(2.40–3.26) | 2.78(187.05) | 2.70(2.32) | 1.42(1.19) |
| Injury, poisoning and procedural complications | Medication error | 171 | < 0.001 | 4.85(4.15–5.68) | 4.82(479.47) | 4.53(3.87) | 2.15(1.92) |
| General disorders and administration site conditions | Local reaction | 158 | < 0.001 | 4.41(3.75–5.18) | 4.38(384.06) | 4.14(3.52) | 2.02(1.78) |
| Eye disorders | Eye movement disorder | 147 | < 0.001 | 4.41(3.73–5.22) | 4.39(358.37) | 4.15(3.51) | 2.02(1.78) |
| Investigations | Blood glucose normal | 144 | < 0.001 | 2.44(2.06–2.88) | 2.43(116.38) | 2.37(2.01) | 1.23(0.99) |
| Nervous system disorders | Dyskinesia | 143 | < 0.001 | 3.44(2.90–4.07) | 3.42(232.14) | 3.29(2.78) | 1.69(1.45) |
| Injury, poisoning and procedural complications | Product administered at inappropriate site | 140 | < 0.001 | 2.97(2.50–3.52) | 2.96(172.97) | 2.86(2.41) | 1.49(1.25) |
| Musculoskeletal and connective tissue disorders | Posture abnormal | 128 | < 0.001 | 7.85(6.52–9.44) | 7.81(670.72) | 7.00(5.82) | 2.74(2.47) |
| Infections and infestations | Injection site cellulitis | 113 | < 0.001 | 3.96(3.27–4.79) | 3.94(232.59) | 3.75(3.10) | 1.87(1.59) |
| General disorders and administration site conditions | Nodule | 110 | < 0.001 | 2.93(2.42–3.55) | 2.92(132.56) | 2.83(2.33) | 1.47(1.19) |
| General disorders and administration site conditions | Injection site oedema | 78 | < 0.001 | 5.36(4.25–6.77) | 5.35(252.75) | 4.98(3.95) | 2.24(1.90) |
| Nervous system disorders | Seizure like phenomena | 78 | < 0.001 | 4.15(3.30–5.22) | 4.14(173.39) | 3.93(3.12) | 1.91(1.58) |
| General disorders and administration site conditions | Injection site discomfort | 62 | < 0.001 | 2.75(2.13–3.55) | 2.75(65.79) | 2.67(2.07) | 1.37(1.00) |
| General disorders and administration site conditions | Injection site vesicles | 61 | < 0.001 | 2.73(2.11–3.53) | 2.72(63.56) | 2.65(2.05) | 1.35(0.98) |
| Nervous system disorders | Hypotonia | 50 | < 0.001 | 3.04(2.29–4.04) | 3.03(64.84) | 2.93(2.21) | 1.48(1.08) |
| Musculoskeletal and connective tissue disorders | Muscle rigidity | 46 | < 0.001 | 5.30(3.92–7.17) | 5.29(146.85) | 4.93(3.65) | 2.17(1.74) |
| Injury, poisoning and procedural complications | Face injury | 45 | < 0.001 | 5.45(4.02–7.40) | 5.44(149.37) | 5.06(3.73) | 2.20(1.76) |
| Musculoskeletal and connective tissue disorders | Limb mass | 42 | < 0.001 | 3.49(2.55–4.76) | 3.48(70.19) | 3.34(2.45) | 1.65(1.20) |
| Nervous system disorders | Generalised tonic-clonic seizure | 41 | < 0.001 | 3.01(2.20–4.12) | 3.00(52.17) | 2.91(2.12) | 1.46(1.01) |
| Investigations | X-ray limb normal | 40 | < 0.001 | 3.84(2.79–5.29) | 3.83(78.63) | 3.66(2.66) | 1.76(1.30) |
| Musculoskeletal and connective tissue disorders | Rotator cuff syndrome | 36 | < 0.001 | 3.19(2.28–4.47) | 3.19(51.30) | 3.08(2.20) | 1.52(1.04) |
| Injury, poisoning and procedural complications | Skin laceration | 36 | < 0.001 | 3.13(2.23–4.37) | 3.12(49.33) | 3.01(2.16) | 1.50(1.01) |
| Respiratory, thoracic and mediastinal disorders | Pharyngeal oedema | 34 | < 0.001 | 3.93(2.78–5.56) | 3.93(69.51) | 3.74(2.64) | 1.77(1.28) |
| Nervous system disorders | Altered state of consciousness | 33 | < 0.001 | 4.20(2.95–5.97) | 4.19(74.83) | 3.98(2.79) | 1.85(1.34) |
| Eye disorders | Mydriasis | 32 | < 0.001 | 3.47(2.43–4.96) | 3.47(53.11) | 3.33(2.33) | 1.61(1.10) |
| Pregnancy, puerperium and perinatal conditions | Delivery | 29 | < 0.001 | 5.09(3.48–7.44) | 5.08(87.53) | 4.76(3.25) | 2.05(1.51) |
| Eye disorders | Eyelid oedema | 24 | < 0.001 | 4.63(3.05–7.01) | 4.62(63.14) | 4.36(2.87) | 1.91(1.32) |
| Investigations | Neurological examination normal | 24 | < 0.001 | 3.94(2.60–5.96) | 3.94(49.25) | 3.75(2.48) | 1.73(1.14) |
| General disorders and administration site conditions | Injection site discharge | 22 | < 0.001 | 4.67(3.02–7.21) | 4.67(58.71) | 4.40(2.85) | 1.90(1.29) |
| Nervous system disorders | Hyporesponsive to stimuli | 21 | < 0.001 | 3.54(2.28–5.49) | 3.53(35.97) | 3.39(2.18) | 1.58(0.95) |
| Infections and infestations | Pertussis | 21 | < 0.001 | 20.17(12.28–33.13) | 20.15(284.37) | 15.25(9.28) | 3.18(2.49) |
| General disorders and administration site conditions | Injection site joint pain | 20 | < 0.001 | 3.44(2.19–5.39) | 3.43(32.59) | 3.30(2.10) | 1.54(0.89) |
| Nervous system disorders | Slow response to stimuli | 20 | < 0.001 | 6.62(4.17–10.51) | 6.61(85.65) | 6.04(3.81) | 2.25(1.59) |
| Surgical and medical procedures | Caesarean section | 19 | < 0.001 | 5.35(3.34–8.56) | 5.35(61.54) | 4.98(3.11) | 2.02(1.35) |
| Immune system disorders | Type iii immune complex mediated reaction | 19 | < 0.001 | 11.24(6.88–18.37) | 11.23(148.64) | 9.59(5.87) | 2.71(2.02) |
| Injury, poisoning and procedural complications | Wrong patient received product | 19 | < 0.001 | 5.80(3.62–9.29) | 5.79(68.57) | 5.36(3.35) | 2.10(1.43) |
| Nervous system disorders | Radiculitis brachial | 17 | < 0.001 | 10.16(6.07–17.01) | 10.15(119.57) | 8.80(5.26) | 2.58(1.85) |
| Psychiatric disorders | Tearfulness | 17 | < 0.001 | 4.79(2.92–7.85) | 4.78(47.05) | 4.50(2.74) | 1.87(1.17) |
| General disorders and administration site conditions | Injection site haematoma | 16 | < 0.001 | 4.15(2.50–6.89) | 4.14(35.65) | 3.94(2.37) | 1.70(0.98) |
| Pregnancy, puerperium and perinatal conditions | Premature delivery | 16 | < 0.001 | 5.86(3.50–9.79) | 5.85(58.55) | 5.41(3.24) | 2.06(1.33) |
| Nervous system disorders | Tonic clonic movements | 16 | < 0.001 | 6.29(3.76–10.54) | 6.29(64.23) | 5.77(3.45) | 2.13(1.40) |
| Nervous system disorders | Consciousness fluctuating | 14 | < 0.001 | 5.66(3.27–9.79) | 5.65(48.89) | 5.24(3.03) | 1.98(1.20) |
| Pregnancy, puerperium and perinatal conditions | Premature labour | 12 | < 0.001 | 4.85(2.69–8.73) | 4.84(33.82) | 4.55(2.53) | 1.78(0.95) |
| Respiratory, thoracic and mediastinal disorders | Snoring | 12 | < 0.001 | 4.91(2.73–8.86) | 4.91(34.50) | 4.61(2.56) | 1.79(0.96) |
| Investigations | Tuberculin test | 12 | < 0.001 | 9.13(4.97–16.77) | 9.12(75.09) | 8.03(4.37) | 2.32(1.47) |
| Infections and infestations | Vaccination site cellulitis | 12 | < 0.001 | 5.17(2.86–9.33) | 5.17(37.04) | 4.83(2.67) | 1.84(1.01) |
| Nervous system disorders | Opisthotonus | 11 | < 0.001 | 5.70(3.07–10.59) | 5.70(38.84) | 5.28(2.84) | 1.90(1.03) |
| Surgical and medical procedures | Suture insertion | 11 | < 0.001 | 4.03(2.19–7.42) | 4.02(23.40) | 3.83(2.08) | 1.57(0.71) |
| General disorders and administration site conditions | Administration site swelling | 10 | < 0.001 | 5.32(2.79–10.17) | 5.32(32.17) | 4.96(2.60) | 1.80(0.89) |
| Pregnancy, puerperium and perinatal conditions | Foetal hypokinesia | 10 | < 0.001 | 8.87(4.56–17.26) | 8.87(60.64) | 7.83(4.03) | 2.20(1.27) |
| Investigations | Prenatal screening test | 10 | < 0.001 | 7.14(3.70–13.77) | 7.14(47.05) | 6.47(3.36) | 2.04(1.12) |
| Pregnancy, puerperium and perinatal conditions | Stillbirth | 10 | < 0.001 | 7.61(3.94–14.70) | 7.60(50.75) | 6.84(3.54) | 2.09(1.17) |
| Pregnancy, puerperium and perinatal conditions | Uterine contractions during pregnancy | 10 | < 0.001 | 6.30(3.28–12.09) | 6.29(40.21) | 5.78(3.01) | 1.94(1.03) |
| Investigations | Bordetella test positive | 9 | < 0.001 | 31.00(13.82–69.56) | 30.99(170.79) | 20.61(9.19) | 2.74(1.67) |
| Pregnancy, puerperium and perinatal conditions | Foetal disorder | 9 | < 0.001 | 12.86(6.25–26.45) | 12.85(80.65) | 10.72(5.21) | 2.37(1.37) |
| Psychiatric disorders | Posturing | 9 | < 0.001 | 8.78(4.36–17.70) | 8.78(53.96) | 7.77(3.85) | 2.14(1.16) |
| Pregnancy, puerperium and perinatal conditions | Premature rupture of membranes | 9 | < 0.001 | 9.76(4.82–19.77) | 9.76(60.63) | 8.51(4.20) | 2.21(1.22) |
| Investigations | Spinal x-ray normal | 9 | < 0.001 | 4.32(2.19–8.50) | 4.32(21.38) | 4.09(2.08) | 1.57(0.62) |
| Product issues | Product distribution issue | 8 | < 0.001 | 4.73(2.30–9.73) | 4.73(21.78) | 4.45(2.17) | 1.60(0.60) |
| Investigations | Ultrasound antenatal screen normal | 8 | < 0.001 | 7.10(3.41–14.79) | 7.10(37.37) | 6.44(3.09) | 1.92(0.90) |
| Injury, poisoning and procedural complications | Wrong patient | 8 | < 0.001 | 4.46(2.17–9.16) | 4.46(19.96) | 4.22(2.05) | 1.55(0.55) |
| Pregnancy, puerperium and perinatal conditions | Neonatal disorder | 7 | < 0.001 | 45.55(16.96–122.31) | 45.53(171.49) | 26.05(9.70) | 2.59(1.34) |
| Musculoskeletal and connective tissue disorders | Bursal fluid accumulation | 6 | < 0.001 | 12.55(5.19–30.31) | 12.54(52.50) | 10.51(4.35) | 2.06(0.85) |
| Injury, poisoning and procedural complications | Foetal exposure during pregnancy | 6 | < 0.001 | 5.24(2.27–12.09) | 5.24(18.91) | 4.89(2.12) | 1.55(0.39) |
| Injury, poisoning and procedural complications | Documented hypersensitivity to administered product | 5 | < 0.001 | 18.30(6.70–49.95) | 18.29(62.28) | 14.18(5.19) | 2.04(0.69) |
| Investigations | Heavy metal test | 5 | < 0.001 | 7.14(2.82–18.07) | 7.14(23.53) | 6.47(2.56) | 1.64(0.36) |
| Nervous system disorders | Hypertonia | 5 | < 0.001 | 5.97(2.38–15.00) | 5.97(18.79) | 5.51(2.20) | 1.53(0.27) |
| Nervous system disorders | Miller fisher syndrome | 5 | < 0.001 | 5.42(2.17–13.55) | 5.42(16.50) | 5.05(2.02) | 1.47(0.21) |
| Musculoskeletal and connective tissue disorders | Muscle oedema | 5 | < 0.001 | 8.61(3.37–22.02) | 8.61(29.31) | 7.63(2.99) | 1.74(0.45) |
| Investigations | Mycoplasma test | 5 | < 0.001 | 5.52(2.21–13.82) | 5.52(16.92) | 5.13(2.05) | 1.48(0.22) |
| Pregnancy, puerperium and perinatal conditions | Premature separation of placenta | 5 | < 0.001 | 5.74(2.29–14.38) | 5.74(17.82) | 5.32(2.12) | 1.51(0.24) |
| Immune system disorders | Rubber sensitivity | 5 | < 0.001 | 11.26(4.32–29.33) | 11.26(39.19) | 9.60(3.69) | 1.86(0.56) |
| Surgical and medical procedures | Supine position | 5 | < 0.001 | 9.76(3.79–25.15) | 9.76(33.68) | 8.51(3.30) | 1.80(0.50) |
| Social circumstances | Blood donor | 4 | < 0.001 | 21.29(6.78–66.87) | 21.29(56.71) | 15.88(5.06) | 1.88(0.36) |
| Blood and lymphatic system disorders | Bone marrow oedema | 4 | < 0.001 | 6.16(2.20–17.27) | 6.16(15.65) | 5.67(2.02) | 1.41(0.01) |
| Investigations | Bordetella test | 4 | < 0.001 | 29.27(8.81–97.23) | 29.27(72.81) | 19.85(5.98) | 1.94(0.39) |
| Musculoskeletal and connective tissue disorders | Bursa disorder | 4 | < 0.001 | 7.10(2.51–20.03) | 7.10(18.68) | 6.44(2.28) | 1.48(0.07) |
| Investigations | Cytogenetic analysis abnormal | 4 | < 0.001 | 9.01(3.14–25.81) | 9.01(24.67) | 7.94(2.77) | 1.59(0.17) |
| Infections and infestations | Erysipelas | 4 | < 0.001 | 23.42(7.34–74.68) | 23.42(61.31) | 17.01(5.33) | 1.90(0.37) |
| Pregnancy, puerperium and perinatal conditions | Gestational diabetes | 4 | < 0.001 | 7.10(2.51–20.03) | 7.10(18.68) | 6.44(2.28) | 1.48(0.07) |
| Investigations | Urine bilirubin decreased | 4 | < 0.001 | 8.67(3.03–24.79) | 8.67(23.65) | 7.68(2.69) | 1.57(0.15) |

Tdap, tetanus, diphtheria, and acellular pertussis; PT, preferred term; SOC, System Organ Class; a, the number of reports; ROR, reporting odds ratio; CI, confidence interval; PRR, proportional reporting ratio; χ2, Chi-squared; EBGM, empirical Bayesian geometric mean; EBGM05, the lower 95 two-sided CI of EBGM; IC, information component; IC025, the lower limit of the 95% two-sided CI of the IC.

# Table S4. Clinical significance rating of subgroup-specific priority signals.

|  | **Opisthotonus** | **Radiculitis Brachial** | **Bursal Fluid Accumulation** |
| --- | --- | --- | --- |
| **Subgroup** | adolescents | non-pregnant females | non-pregnant females |
| **Signal strength** | ROR: 8.55(3.6-20.29) | ROR: 14.42(7.71-27) | ROR: 21.22(7.71-58.4) |
| **Key case-review findings** | Median onset: 1 days;  Serious: 1/8;  Recovere: 5/9 | Median onset: 1 days;  Serious: 3/12;  Recovere: 0/12 | Median onset: 1 days;  Serious: 1/5;  Recovere: 0/5 |
| **Main rationale** | clear disproportionality,  consistent temporal pattern, but generally non-serious and self-limited | clear disproportionality, serious outcomes, and consistent temporal pattern | clear disproportionality, serious outcomes, and consistent temporal pattern |
| **Clinical Significance** | Low | High | High |

Clinical significance was rated as High, Moderate, or Low based on the overall review of signal strength and clinical features from case assessment, including seriousness, temporal plausibility, and potential clinical consequences. The rating was intended to support signal prioritization and summary presentation only, and not to imply causality or quantify the true risk.

# Table S5. Signal strength of Tdap vaccine-related positive PT signals and their corresponding SOCs in adolescents aged 10–18 years.

| SOC | PT | a | p–adjust | ROR(95%CI) | PRR(X²) | EBGM(EBGM05) | IC(IC025) |
| --- | --- | --- | --- | --- | --- | --- | --- |
| General disorders and administration site conditions | Injection site erythema | 850 | < 0.001 | 5.20(4.78–5.66) | 4.64(1793.05) | 3.59(3.30) | 1.84(1.72) |
| General disorders and administration site conditions | Injection site swelling | 675 | < 0.001 | 4.97(4.53–5.46) | 4.55(1377.47) | 3.54(3.22) | 1.82(1.69) |
| Skin and subcutaneous tissue disorders | Erythema | 567 | < 0.001 | 4.04(3.66–4.46) | 3.77(892.53) | 3.08(2.79) | 1.62(1.48) |
| General disorders and administration site conditions | Injection site warmth | 387 | < 0.001 | 5.23(4.63–5.91) | 4.98(869.80) | 3.77(3.33) | 1.90(1.73) |
| General disorders and administration site conditions | Peripheral swelling | 326 | < 0.001 | 3.92(3.45–4.46) | 3.77(507.78) | 3.08(2.71) | 1.61(1.44) |
| Skin and subcutaneous tissue disorders | Skin warm | 302 | < 0.001 | 4.91(4.29–5.63) | 4.73(635.55) | 3.63(3.17) | 1.85(1.66) |
| General disorders and administration site conditions | Swelling | 207 | < 0.001 | 3.18(2.72–3.72) | 3.11(235.41) | 2.66(2.27) | 1.39(1.18) |
| General disorders and administration site conditions | Injection site pruritus | 186 | < 0.001 | 4.76(4.01–5.66) | 4.65(381.76) | 3.59(3.02) | 1.82(1.58) |
| General disorders and administration site conditions | Injection site induration | 161 | < 0.001 | 5.30(4.39–6.40) | 5.19(376.63) | 3.88(3.21) | 1.92(1.67) |
| Infections and infestations | Cellulitis | 104 | < 0.001 | 7.19(5.62–9.20) | 7.09(336.32) | 4.75(3.72) | 2.19(1.86) |
| General disorders and administration site conditions | Injection site rash | 85 | < 0.001 | 3.06(2.41–3.89) | 3.03(91.93) | 2.61(2.05) | 1.34(1.01) |
| Musculoskeletal and connective tissue disorders | Posture abnormal | 83 | < 0.001 | 3.93(3.06–5.04) | 3.89(133.26) | 3.15(2.45) | 1.61(1.26) |
| General disorders and administration site conditions | Injection site reaction | 77 | < 0.001 | 3.21(2.49–4.14) | 3.19(90.69) | 2.71(2.10) | 1.40(1.04) |
| General disorders and administration site conditions | Induration | 72 | < 0.001 | 4.57(3.47–6.01) | 4.53(142.04) | 3.52(2.68) | 1.76(1.38) |
| General disorders and administration site conditions | Local reaction | 64 | < 0.001 | 4.01(3.01–5.34) | 3.98(106.17) | 3.21(2.41) | 1.62(1.22) |
| General disorders and administration site conditions | Injection site urticaria | 55 | < 0.001 | 3.84(2.83–5.22) | 3.82(85.88) | 3.11(2.29) | 1.57(1.14) |
| Infections and infestations | Injection site cellulitis | 41 | < 0.001 | 5.65(3.88–8.22) | 5.62(104.47) | 4.09(2.81) | 1.91(1.40) |
| General disorders and administration site conditions | Vaccination site swelling | 34 | < 0.001 | 3.60(2.44–5.29) | 3.58(48.25) | 2.96(2.01) | 1.46(0.93) |
| General disorders and administration site conditions | Injection site oedema | 33 | < 0.001 | 5.72(3.76–8.69) | 5.69(85.18) | 4.13(2.72) | 1.89(1.33) |
| Nervous system disorders | Slow response to stimuli | 14 | < 0.001 | 5.32(2.82–10.04) | 5.31(33.43) | 3.94(2.09) | 1.66(0.81) |
| General disorders and administration site conditions | Injection site irritation | 10 | < 0.001 | 8.14(3.61–18.34) | 8.13(36.49) | 5.16(2.29) | 1.83(0.78) |
| Nervous system disorders | Opisthotonus | 9 | < 0.001 | 8.55(3.60–20.29) | 8.54(34.23) | 5.31(2.24) | 1.81(0.71) |
| Immune system disorders | Type iii immune complex mediated reaction | 6 | < 0.001 | 17.09(4.82–60.57) | 17.07(36.32) | 7.43(2.10) | 1.85(0.44) |

Tdap, tetanus, diphtheria, and acellular pertussis; PT, preferred term; SOC, System Organ Class; a, the number of reports; ROR, reporting odds ratio; CI, confidence interval; PRR, proportional reporting ratio; χ2, Chi-squared; EBGM, empirical Bayesian geometric mean; EBGM05, the lower 95 two-sided CI of EBGM; IC, information component; IC025, the lower limit of the 95% two-sided CI of the IC.

# Table S6. Signal strength of Tdap vaccine-related positive PT signals and their corresponding SOCs in older adults aged ≥65 years.

| SOC | PT | a | p–adjust | ROR(95%CI) | PRR(X²) | EBGM(EBGM05) | IC(IC025) |
| --- | --- | --- | --- | --- | --- | --- | --- |
| General disorders and administration site conditions | Injection site erythema | 354 | < 0.001 | 3.18(2.84–3.58) | 2.79(426.62) | 2.76(2.45) | 1.45(1.29) |
| General disorders and administration site conditions | Injection site swelling | 263 | < 0.001 | 3.08(2.70–3.51) | 2.80(313.32) | 2.76(2.42) | 1.45(1.27) |
| General disorders and administration site conditions | Injection site warmth | 130 | < 0.001 | 2.60(2.17–3.11) | 2.49(117.66) | 2.47(2.06) | 1.28(1.03) |
| General disorders and administration site conditions | Injected limb mobility decreased | 58 | < 0.001 | 3.76(2.89–4.90) | 3.68(111.37) | 3.62(2.78) | 1.78(1.40) |
| Injury, poisoning and procedural complications | Product administered to patient of inappropriate age | 50 | < 0.001 | 17.43(12.96–23.42) | 17.00(678.24) | 15.39(11.45) | 3.57(3.15) |
| General disorders and administration site conditions | Injection site induration | 39 | < 0.001 | 2.89(2.10–3.98) | 2.85(46.30) | 2.82(2.04) | 1.41(0.95) |
| General disorders and administration site conditions | Injection site bruising | 37 | < 0.001 | 3.05(2.20–4.24) | 3.01(49.13) | 2.97(2.14) | 1.48(1.01) |
| Injury, poisoning and procedural complications | Wrong product administered | 34 | < 0.001 | 3.21(2.28–4.52) | 3.17(49.82) | 3.13(2.22) | 1.54(1.05) |
| General disorders and administration site conditions | Injection site mass | 32 | < 0.001 | 4.44(3.11–6.33) | 4.38(81.51) | 4.29(3.01) | 1.94(1.43) |
| Musculoskeletal and connective tissue disorders | Joint range of motion decreased | 21 | < 0.001 | 3.76(2.43–5.80) | 3.73(40.99) | 3.66(2.37) | 1.67(1.05) |
| General disorders and administration site conditions | Injection site nodule | 19 | < 0.001 | 5.30(3.35–8.39) | 5.26(63.42) | 5.11(3.23) | 2.05(1.39) |
| Injury, poisoning and procedural complications | Product administered at inappropriate site | 19 | < 0.001 | 3.62(2.29–5.72) | 3.59(34.85) | 3.53(2.24) | 1.61(0.96) |
| General disorders and administration site conditions | Local reaction | 18 | < 0.001 | 5.82(3.62–9.33) | 5.77(68.51) | 5.60(3.49) | 2.13(1.46) |
| General disorders and administration site conditions | Injection site urticaria | 16 | < 0.001 | 4.41(2.68–7.27) | 4.39(40.74) | 4.29(2.61) | 1.80(1.09) |
| Infections and infestations | Injection site cellulitis | 14 | < 0.001 | 4.94(2.90–8.44) | 4.92(42.36) | 4.79(2.81) | 1.89(1.13) |
| Injury, poisoning and procedural complications | Medication error | 12 | < 0.001 | 9.46(5.27–16.98) | 9.41(84.97) | 8.92(4.97) | 2.41(1.59) |
| Investigations | Electromyogram | 8 | < 0.001 | 5.55(2.74–11.25) | 5.53(28.65) | 5.37(2.65) | 1.77(0.78) |
| Musculoskeletal and connective tissue disorders | Rotator cuff syndrome | 7 | < 0.001 | 5.13(2.41–10.92) | 5.12(22.45) | 4.98(2.34) | 1.64(0.59) |
| General disorders and administration site conditions | Injection site haematoma | 6 | < 0.001 | 11.11(4.84–25.47) | 11.07(51.25) | 10.39(4.53) | 2.05(0.90) |
| General disorders and administration site conditions | Injection site irritation | 6 | < 0.001 | 4.64(2.06–10.48) | 4.63(16.60) | 4.53(2.01) | 1.48(0.36) |
| Product issues | Needle issue | 5 | < 0.001 | 6.89(2.81–16.91) | 6.88(24.04) | 6.62(2.70) | 1.65(0.42) |
| Injury, poisoning and procedural complications | Vaccination error | 5 | < 0.001 | 8.92(3.62–22.01) | 8.90(33.14) | 8.46(3.43) | 1.79(0.55) |
| Investigations | Immunology test | 4 | < 0.001 | 7.31(2.68–19.95) | 7.29(20.73) | 7.00(2.57) | 1.52(0.16) |
| Infections and infestations | Pertussis | 3 | < 0.001 | 90.94(21.72–380.82) | 90.81(166.54) | 57.13(13.64) | 1.81(0.02) |

Tdap, tetanus, diphtheria, and acellular pertussis; PT, preferred term; SOC, System Organ Class; a, the number of reports; ROR, reporting odds ratio; CI, confidence interval; PRR, proportional reporting ratio; χ2, Chi-squared; EBGM, empirical Bayesian geometric mean; EBGM05, the lower 95 two-sided CI of EBGM; IC, information component; IC025, the lower limit of the 95% two-sided CI of the IC.

# Table S7. Clinical case-by-case review for radiculitis brachial signal in non-pregnant female subgroup.

|  | **Age**  **(years)** | **Time to onset**  **(days)** | **Clinical features** | **Features compatible with classical brachial neuritis** | **Diagnostic basis** | **Diagnostic workup** | **Treatment** | **Final outcome** |
| --- | --- | --- | --- | --- | --- | --- | --- | --- |
| 1 | 47 | 1 | Pain in left arm after Tdap vaccination-worsened by next morning.  Day 41 post-vaccination: persistent pain and weakness in left arm | Yes | Diagnosed at primary care doctor 27 days post-vaccination. | Unknown | Has had 3 physical therapy sessions | Not recovered at reporting |
| 2 | 54 | 15 | Two weeks post vaccine tingling, transverse (L) ring finger, decreased power of left shoulder and arm. | Yes | Unknown | Reported diagnosis: Dx: (L) brachial neuritis | Unknown | Not recovered at reporting |
| 3 | 58 | 0 | Brachial Neuritis left arm with pain that woke her from sleep the night after receiving the vaccine. Gradually improved over the next week. | No | Unknown | Unknown | Unknown | Not recovered at reporting |
| 4 | 64 | 2 | Burning/stinging/numbing pain in arms , elbows, wrists, hands and knees; weakness of the arms, wrists, and elbows. | Yes | Unknown | Unknown | Unknown | Not recovered at reporting |
| 5 | 61 | 0 | Within hours very painful where shot was given;  the next night: Severe right arm pain; untouchable due to pain; active movement lost;  Day 4 post-vaccination: Right hand ice cold, numb, tingling; complete right-sided immobility. | Yes | Diagnosed by regular doctor | Unknown | Prednisone;  doing exercises | Not recovered at reporting |
| 6 | 36 | 0 | Numbness and tingling in arm around injection site and extending into the right hand, muscle weakness | Yes | Unknown | Unknown | Unknown | Unknown |
| 7 | 49 | 1 | Started the following day with a sore upper arm, then quickly progressed to diffused shoulder, neck, upper back, chest pain that developed into burning, tingling and numbing sensations from the left, upper arm down to the elbow, then into the hand. | Yes | Diagnosed by Primary Care Physician | Multi-modality, medical assessments Imaging has been discussed.  Specific results not provided | Medication prescribed (unspecified);  acupuncture, massage and chiropractic care | Not recovered at reporting |
| 8 | 41 | 1 | Night post-tetanus shot: Severe neck/shoulder/arm pain; ADL impairment due to pain; 2-finger numbness.  Following week: Persistent severe pain; ongoing arm weakness | Yes | Diagnosed by neurologist | had an MRI and electrodiagnostic evaluation to confirm. During the electrodiagnostic evaluation, muscle atrophy was noted. | Steroids, gabapentin | Not recovered at reporting |
| 9 | 44 | 1 | Day 1 post-vaccination: arm swelling, tenderness, deep ache, and entire body aches.  Day 6 post-vaccination: new-onset radiating pain; decreased ROM.  Day 28 post-vaccination: persistent constant throbbing, decreased ROM, radiating left shoulder pain; ADL impairment. | Yes | Diagnosed by doctor | Unknown | Unknown | Not recovered at reporting |
| 10 | 75 | 2 | Days post-vaccination: Large hard lump and bruise at injection site; bilateral arm pain; muscle weakness. | Yes | Diagnosed by doctor | had an MRI evaluation;  Specific results not provided | gabapentin | Not recovered at reporting |
| 11 | 48 | 3 | Unknown | Unknown | Unknown | Unknown | physical therapy | Not recovered at reporting |
| 12 | 65 | Unknown | Numbness; back pain | Yes | The neurologist suspect it could be brachial neuritis. | Unknown | Unknown | Unknown |

# Table S8. Clinical details of subgroup-specific priority signals.

|  | **Opisthotonus**  **in adolescents** | **Radiculitis Brachial**  **in non-pregnant females** | **Bursal Fluid Accumulation**  **in non-pregnant females** |
| --- | --- | --- | --- |
| **Total number** | 9 | 12 | 5 |
| **Age in years, median (range)** | 12 (10–18) | 51.5 (36–75) | 44 (37–52) |
| **Onset time in days, median (range)** | 1 (0–9) | 1 (0–15) | 1 (0–1) |
| **Gender** | |  |  |
| Male | 5 | 0 | 0 |
| Female | 4 | 12 | 5 |
| **Alone** | |  |  |
| Yes | 2 | 12 | 4 |
| No | 7 | 0 | 1 |
| **Serious** | |  |  |
| Yes | 1 (hospitalized: 1) | 3 (disability: 3) | 1 (disability: 1) |
| No | 8 | 9 | 4 |
| **Recovered** | |  |  |
| Yes | 5 | 0 | 0 |
| No | 0 | 10 | 3 |
| Unknown | 4 | 2 | 2 |
| **Concomitant symptoms** | Eye Movement Disorder (5) | Pain in Extremity (7) | Joint Range of Motion Decreased (2) |
|  | Loss of Consciousness (4) | Muscular Weakness (6) | Hypoaesthesia (2) |
|  | Pallor (4) | Hypoaesthesia (6) | Magnetic Resonance Imaging Abnormal (2) |

# Table S9. Signal strength of Tdap vaccine-related positive PT signals and their corresponding SOCs in male.

| SOC | PT | a | p–adjust | ROR(95%CI) | PRR(X²) | EBGM(EBGM05) | IC(IC025) |
| --- | --- | --- | --- | --- | --- | --- | --- |
| General disorders and administration site conditions | Injection site erythema | 814 | < 0.001 | 4.35(4.03–4.69) | 3.97(1731.49) | 3.76(3.48) | 1.90(1.80) |
| General disorders and administration site conditions | Injection site swelling | 630 | < 0.001 | 3.93(3.61–4.27) | 3.67(1171.57) | 3.49(3.21) | 1.80(1.68) |
| Skin and subcutaneous tissue disorders | Erythema | 575 | < 0.001 | 3.64(3.33–3.97) | 3.43(948.30) | 3.27(3.00) | 1.70(1.58) |
| Nervous system disorders | Syncope | 546 | < 0.001 | 3.25(2.97–3.55) | 3.08(740.11) | 2.96(2.70) | 1.56(1.43) |
| Injury, poisoning and procedural complications | Wrong product administered | 483 | < 0.001 | 5.62(5.10–6.19) | 5.31(1551.11) | 4.90(4.45) | 2.28(2.14) |
| Vascular disorders | Pallor | 393 | < 0.001 | 4.69(4.21–5.21) | 4.49(990.45) | 4.20(3.78) | 2.06(1.91) |
| Nervous system disorders | Loss of consciousness | 382 | < 0.001 | 3.04(2.73–3.37) | 2.93(467.14) | 2.82(2.54) | 1.49(1.34) |
| General disorders and administration site conditions | Injection site warmth | 357 | < 0.001 | 4.79(4.29–5.36) | 4.61(934.74) | 4.31(3.85) | 2.09(1.93) |
| General disorders and administration site conditions | Peripheral swelling | 349 | < 0.001 | 2.77(2.48–3.09) | 2.68(355.97) | 2.60(2.33) | 1.37(1.21) |
| Skin and subcutaneous tissue disorders | Skin warm | 246 | < 0.001 | 4.49(3.94–5.13) | 4.38(594.49) | 4.11(3.60) | 2.02(1.83) |
| General disorders and administration site conditions | Swelling | 221 | < 0.001 | 2.68(2.34–3.08) | 2.63(215.20) | 2.55(2.22) | 1.34(1.14) |
| General disorders and administration site conditions | Immediate post-injection reaction | 174 | < 0.001 | 3.31(2.84–3.87) | 3.26(257.40) | 3.12(2.67) | 1.62(1.40) |
| General disorders and administration site conditions | Injection site pruritus | 160 | < 0.001 | 3.10(2.64–3.65) | 3.06(210.37) | 2.94(2.50) | 1.53(1.30) |
| General disorders and administration site conditions | Injection site induration | 133 | < 0.001 | 4.53(3.79–5.42) | 4.47(330.30) | 4.19(3.50) | 2.03(1.77) |
| General disorders and administration site conditions | Injected limb mobility decreased | 114 | < 0.001 | 3.81(3.14–4.61) | 3.76(216.30) | 3.57(2.95) | 1.80(1.52) |
| Infections and infestations | Cellulitis | 113 | < 0.001 | 5.26(4.33–6.40) | 5.20(348.48) | 4.81(3.95) | 2.21(1.93) |
| General disorders and administration site conditions | Tenderness | 112 | < 0.001 | 3.13(2.58–3.80) | 3.10(150.80) | 2.98(2.46) | 1.54(1.27) |
| Injury, poisoning and procedural complications | Head injury | 100 | < 0.001 | 3.46(2.82–4.24) | 3.43(161.66) | 3.27(2.67) | 1.67(1.38) |
| Nervous system disorders | Presyncope | 89 | < 0.001 | 2.91(2.34–3.60) | 2.88(103.95) | 2.78(2.24) | 1.44(1.13) |
| Injury, poisoning and procedural complications | Medication error | 66 | < 0.001 | 4.37(3.39–5.62) | 4.34(156.50) | 4.07(3.17) | 1.95(1.59) |
| Eye disorders | Eye movement disorder | 65 | < 0.001 | 3.62(2.81–4.66) | 3.60(114.18) | 3.43(2.66) | 1.71(1.35) |
| Nervous system disorders | Dyskinesia | 63 | < 0.001 | 3.48(2.69–4.49) | 3.45(103.09) | 3.30(2.55) | 1.66(1.29) |
| Musculoskeletal and connective tissue disorders | Posture abnormal | 63 | < 0.001 | 7.34(5.63–9.57) | 7.29(299.10) | 6.50(4.98) | 2.57(2.19) |
| General disorders and administration site conditions | Induration | 62 | < 0.001 | 4.73(3.64–6.14) | 4.70(165.49) | 4.38(3.38) | 2.04(1.67) |
| General disorders and administration site conditions | Local reaction | 55 | < 0.001 | 4.76(3.60–6.28) | 4.73(148.11) | 4.41(3.34) | 2.04(1.64) |
| General disorders and administration site conditions | Injection site urticaria | 41 | < 0.001 | 3.72(2.70–5.11) | 3.70(75.43) | 3.52(2.56) | 1.71(1.26) |
| Nervous system disorders | Seizure like phenomena | 41 | < 0.001 | 3.98(2.89–5.47) | 3.96(84.35) | 3.75(2.72) | 1.80(1.34) |
| Infections and infestations | Injection site cellulitis | 33 | < 0.001 | 4.42(3.09–6.31) | 4.40(79.89) | 4.13(2.89) | 1.90(1.39) |
| General disorders and administration site conditions | Injection site oedema | 32 | < 0.001 | 6.04(4.18–8.71) | 6.01(119.63) | 5.48(3.80) | 2.25(1.72) |
| Injury, poisoning and procedural complications | Face injury | 25 | < 0.001 | 5.68(3.76–8.60) | 5.67(86.46) | 5.20(3.43) | 2.13(1.54) |
| General disorders and administration site conditions | Injection site haemorrhage | 24 | < 0.001 | 3.65(2.41–5.53) | 3.64(42.97) | 3.47(2.29) | 1.63(1.04) |
| General disorders and administration site conditions | Injection site vesicles | 23 | < 0.001 | 3.48(2.28–5.31) | 3.47(37.89) | 3.31(2.17) | 1.56(0.96) |
| General disorders and administration site conditions | Injection site discomfort | 22 | < 0.001 | 3.31(2.15–5.09) | 3.30(33.14) | 3.16(2.05) | 1.50(0.88) |
| Musculoskeletal and connective tissue disorders | Muscle rigidity | 17 | < 0.001 | 3.99(2.43–6.53) | 3.98(35.16) | 3.76(2.29) | 1.66(0.96) |
| Nervous system disorders | Altered state of consciousness | 13 | < 0.001 | 3.72(2.12–6.53) | 3.71(24.02) | 3.53(2.01) | 1.52(0.73) |
| Musculoskeletal and connective tissue disorders | Limb mass | 13 | < 0.001 | 5.53(3.12–9.81) | 5.52(43.42) | 5.08(2.86) | 1.92(1.11) |
| Investigations | Magnetic resonance imaging spinal normal | 12 | < 0.001 | 4.10(2.28–7.39) | 4.10(26.01) | 3.87(2.15) | 1.60(0.77) |
| Injury, poisoning and procedural complications | Wrong patient received product | 11 | < 0.001 | 6.71(3.58–12.59) | 6.70(47.11) | 6.03(3.21) | 2.02(1.14) |
| Nervous system disorders | Slow response to stimuli | 8 | < 0.001 | 4.88(2.36–10.08) | 4.87(22.46) | 4.53(2.19) | 1.62(0.60) |
| Nervous system disorders | Tonic clonic movements | 8 | < 0.001 | 6.32(3.03–13.19) | 6.32(31.84) | 5.73(2.75) | 1.83(0.80) |
| Immune system disorders | Type iii immune complex mediated reaction | 8 | < 0.001 | 11.91(5.51–25.73) | 11.89(64.63) | 9.82(4.54) | 2.23(1.17) |
| Infections and infestations | Pertussis | 7 | < 0.001 | 17.71(7.49–41.89) | 17.69(81.67) | 13.36(5.65) | 2.31(1.14) |
| Surgical and medical procedures | Suture insertion | 7 | < 0.001 | 5.06(2.33–11.01) | 5.05(20.70) | 4.69(2.15) | 1.59(0.51) |
| Psychiatric disorders | Tearfulness | 7 | < 0.001 | 13.62(5.91–31.39) | 13.61(64.44) | 10.93(4.74) | 2.20(1.06) |
| Nervous system disorders | Opisthotonus | 6 | < 0.001 | 5.14(2.22–11.92) | 5.14(18.17) | 4.76(2.05) | 1.52(0.36) |
| Investigations | Tuberculin test | 6 | < 0.001 | 13.80(5.59–34.04) | 13.79(55.91) | 11.05(4.48) | 2.08(0.85) |
| Investigations | Bordetella test positive | 5 | < 0.001 | 63.23(16.98–235.52) | 63.19(136.01) | 28.64(7.69) | 2.28(0.77) |
| Nervous system disorders | Demyelinating polyneuropathy | 5 | < 0.001 | 6.17(2.44–15.61) | 6.16(19.29) | 5.60(2.21) | 1.54(0.27) |
| Nervous system disorders | Hypertonia | 5 | < 0.001 | 12.65(4.74–33.70) | 12.64(42.87) | 10.31(3.87) | 1.90(0.57) |
| General disorders and administration site conditions | Injection site macule | 5 | < 0.001 | 8.72(3.37–22.54) | 8.72(29.13) | 7.58(2.93) | 1.73(0.44) |
| Investigations | Mononucleosis heterophile test | 5 | < 0.001 | 6.66(2.62–16.91) | 6.65(21.22) | 5.99(2.36) | 1.59(0.31) |
| Investigations | Spinal x-ray normal | 5 | < 0.001 | 7.02(2.76–17.91) | 7.02(22.67) | 6.29(2.47) | 1.62(0.34) |
| Infections and infestations | Vaccination site cellulitis | 5 | < 0.001 | 9.37(3.61–24.33) | 9.36(31.51) | 8.05(3.10) | 1.77(0.47) |
| General disorders and administration site conditions | Administration site swelling | 4 | < 0.001 | 7.49(2.62–21.42) | 7.49(19.59) | 6.65(2.33) | 1.50(0.08) |
| Infections and infestations | Meningitis aseptic | 4 | < 0.001 | 7.49(2.62–21.42) | 7.49(19.59) | 6.65(2.33) | 1.50(0.08) |
| Investigations | Mycoplasma test | 4 | < 0.001 | 6.74(2.37–19.15) | 6.74(17.25) | 6.06(2.14) | 1.45(0.03) |
| Product issues | Product distribution issue | 4 | < 0.001 | 7.22(2.53–20.60) | 7.22(18.76) | 6.44(2.26) | 1.48(0.06) |

Tdap, tetanus, diphtheria, and acellular pertussis; PT, preferred term; SOC, System Organ Class; a, the number of reports; ROR, reporting odds ratio; CI, confidence interval; PRR, proportional reporting ratio; χ2, Chi-squared; EBGM, empirical Bayesian geometric mean; EBGM05, the lower 95 two-sided CI of EBGM; IC, information component; IC025, the lower limit of the 95% two-sided CI of the IC.

# Table S10. Signal strength of Tdap vaccine-related positive PT signals and their corresponding SOCs in non-pregnant females.

| SOC | PT | a | p–adjust | ROR(95%CI) | PRR(X²) | EBGM(EBGM05) | IC(IC025) |
| --- | --- | --- | --- | --- | --- | --- | --- |
| General disorders and administration site conditions | Injection site erythema | 1772 | < 0.001 | 2.48(2.36–2.61) | 2.25(1280.33) | 2.21(2.10) | 1.14(1.07) |
| General disorders and administration site conditions | Injection site swelling | 1452 | < 0.001 | 2.51(2.38–2.66) | 2.32(1117.20) | 2.28(2.15) | 1.18(1.10) |
| General disorders and administration site conditions | Injection site warmth | 825 | < 0.001 | 2.31(2.15–2.48) | 2.21(548.97) | 2.17(2.02) | 1.12(1.01) |
| Nervous system disorders | Syncope | 652 | < 0.001 | 3.53(3.25–3.83) | 3.39(1058.98) | 3.26(3.01) | 1.70(1.58) |
| Skin and subcutaneous tissue disorders | Skin warm | 550 | < 0.001 | 2.42(2.21–2.64) | 2.35(419.42) | 2.30(2.11) | 1.20(1.07) |
| Injury, poisoning and procedural complications | Wrong product administered | 522 | < 0.001 | 6.51(5.94–7.14) | 6.26(2117.91) | 5.79(5.28) | 2.52(2.39) |
| Nervous system disorders | Loss of consciousness | 429 | < 0.001 | 3.18(2.88–3.51) | 3.10(589.24) | 3.00(2.72) | 1.58(1.44) |
| Vascular disorders | Pallor | 388 | < 0.001 | 5.64(5.07–6.26) | 5.48(1317.36) | 5.13(4.61) | 2.34(2.19) |
| General disorders and administration site conditions | Injection site induration | 352 | < 0.001 | 2.88(2.58–3.21) | 2.82(400.34) | 2.74(2.46) | 1.45(1.29) |
| General disorders and administration site conditions | Injected limb mobility decreased | 295 | < 0.001 | 3.49(3.10–3.94) | 3.43(485.85) | 3.31(2.94) | 1.71(1.54) |
| Injury, poisoning and procedural complications | Fall | 229 | < 0.001 | 2.45(2.14–2.80) | 2.42(185.16) | 2.37(2.07) | 1.23(1.04) |
| Infections and infestations | Cellulitis | 195 | < 0.001 | 3.46(2.99–4.00) | 3.42(318.13) | 3.29(2.85) | 1.70(1.49) |
| General disorders and administration site conditions | Injection site mass | 165 | < 0.001 | 2.56(2.19–2.99) | 2.53(148.20) | 2.48(2.12) | 1.29(1.06) |
| General disorders and administration site conditions | Injection site nodule | 145 | < 0.001 | 4.60(3.88–5.45) | 4.55(376.20) | 4.32(3.64) | 2.07(1.83) |
| General disorders and administration site conditions | Induration | 137 | < 0.001 | 2.75(2.31–3.26) | 2.73(144.14) | 2.65(2.23) | 1.39(1.14) |
| Musculoskeletal and connective tissue disorders | Musculoskeletal pain | 133 | < 0.001 | 3.20(2.69–3.81) | 3.17(189.43) | 3.07(2.58) | 1.59(1.34) |
| Musculoskeletal and connective tissue disorders | Joint range of motion decreased | 118 | < 0.001 | 2.80(2.33–3.37) | 2.78(129.36) | 2.71(2.25) | 1.41(1.14) |
| Injury, poisoning and procedural complications | Head injury | 103 | < 0.001 | 3.94(3.23–4.81) | 3.91(210.95) | 3.74(3.07) | 1.86(1.57) |
| General disorders and administration site conditions | Local reaction | 99 | < 0.001 | 4.43(3.61–5.44) | 4.40(244.00) | 4.18(3.41) | 2.01(1.72) |
| Injury, poisoning and procedural complications | Product administered at inappropriate site | 94 | < 0.001 | 3.00(2.44–3.69) | 2.98(118.74) | 2.89(2.35) | 1.50(1.20) |
| General disorders and administration site conditions | Nodule | 84 | < 0.001 | 3.06(2.46–3.82) | 3.05(110.56) | 2.95(2.37) | 1.52(1.21) |
| Eye disorders | Eye movement disorder | 80 | < 0.001 | 5.00(3.98–6.28) | 4.97(235.78) | 4.68(3.73) | 2.15(1.83) |
| Nervous system disorders | Dyskinesia | 78 | < 0.001 | 3.38(2.69–4.25) | 3.37(123.41) | 3.25(2.58) | 1.65(1.32) |
| Infections and infestations | Injection site cellulitis | 74 | < 0.001 | 3.83(3.03–4.85) | 3.81(145.15) | 3.65(2.89) | 1.81(1.47) |
| Injury, poisoning and procedural complications | Medication error | 66 | < 0.001 | 4.92(3.83–6.33) | 4.90(190.51) | 4.62(3.60) | 2.12(1.76) |
| Musculoskeletal and connective tissue disorders | Posture abnormal | 63 | < 0.001 | 7.84(6.03–10.19) | 7.80(332.95) | 7.06(5.43) | 2.68(2.30) |
| General disorders and administration site conditions | Injection site oedema | 45 | < 0.001 | 4.96(3.66–6.72) | 4.95(131.56) | 4.66(3.44) | 2.09(1.66) |
| Nervous system disorders | Seizure like phenomena | 37 | < 0.001 | 4.19(3.00–5.84) | 4.17(83.90) | 3.98(2.85) | 1.86(1.39) |
| Investigations | X-ray limb | 33 | < 0.001 | 3.18(2.24–4.51) | 3.17(46.80) | 3.07(2.16) | 1.51(1.01) |
| Investigations | X-ray limb normal | 30 | < 0.001 | 4.44(3.06–6.43) | 4.43(74.52) | 4.21(2.90) | 1.91(1.38) |
| Musculoskeletal and connective tissue disorders | Limb mass | 29 | < 0.001 | 3.23(2.22–4.70) | 3.23(42.44) | 3.12(2.15) | 1.52(0.98) |
| Musculoskeletal and connective tissue disorders | Muscle rigidity | 29 | < 0.001 | 6.54(4.46–9.58) | 6.52(123.01) | 6.01(4.10) | 2.34(1.79) |
| Musculoskeletal and connective tissue disorders | Rotator cuff syndrome | 28 | < 0.001 | 3.63(2.48–5.31) | 3.62(50.31) | 3.48(2.38) | 1.65(1.11) |
| Nervous system disorders | Hypotonia | 27 | < 0.001 | 3.40(2.31–5.01) | 3.40(43.35) | 3.27(2.22) | 1.57(1.02) |
| Respiratory, thoracic and mediastinal disorders | Pharyngeal oedema | 25 | < 0.001 | 4.10(2.73–6.14) | 4.09(54.86) | 3.90(2.60) | 1.78(1.21) |
| Nervous system disorders | Generalised tonic-clonic seizure | 23 | < 0.001 | 3.52(2.31–5.35) | 3.51(39.15) | 3.38(2.22) | 1.59(0.99) |
| Eye disorders | Mydriasis | 21 | < 0.001 | 4.06(2.61–6.31) | 4.05(45.36) | 3.87(2.49) | 1.74(1.11) |
| Nervous system disorders | Altered state of consciousness | 20 | < 0.001 | 4.65(2.95–7.33) | 4.65(53.34) | 4.40(2.79) | 1.88(1.24) |
| Injury, poisoning and procedural complications | Face injury | 20 | < 0.001 | 5.00(3.17–7.88) | 4.99(59.21) | 4.70(2.98) | 1.96(1.31) |
| Injury, poisoning and procedural complications | Skin laceration | 19 | < 0.001 | 3.46(2.18–5.49) | 3.45(31.45) | 3.33(2.10) | 1.54(0.88) |
| Infections and infestations | Injection site infection | 18 | < 0.001 | 3.36(2.09–5.40) | 3.36(28.34) | 3.24(2.02) | 1.50(0.82) |
| Eye disorders | Eyelid oedema | 17 | < 0.001 | 5.18(3.16–8.50) | 5.18(52.99) | 4.86(2.96) | 1.96(1.26) |
| General disorders and administration site conditions | Injection site joint pain | 17 | < 0.001 | 4.73(2.89–7.74) | 4.72(46.48) | 4.47(2.73) | 1.86(1.16) |
| Infections and infestations | Rash pustular | 15 | < 0.001 | 3.63(2.16–6.11) | 3.63(27.04) | 3.49(2.07) | 1.55(0.81) |
| Nervous system disorders | Hyporesponsive to stimuli | 14 | < 0.001 | 5.34(3.09–9.22) | 5.33(45.51) | 5.00(2.90) | 1.93(1.16) |
| Nervous system disorders | Radiculitis brachial | 12 | < 0.001 | 14.42(7.71–27.00) | 14.41(122.10) | 11.93(6.38) | 2.64(1.77) |
| Nervous system disorders | Slow response to stimuli | 12 | < 0.001 | 8.40(4.60–15.34) | 8.39(69.04) | 7.53(4.12) | 2.27(1.42) |
| General disorders and administration site conditions | Injection site discharge | 11 | < 0.001 | 4.17(2.27–7.68) | 4.17(24.85) | 3.97(2.16) | 1.61(0.75) |
| General disorders and administration site conditions | Injection site haematoma | 11 | < 0.001 | 4.19(2.28–7.72) | 4.19(25.09) | 3.99(2.17) | 1.61(0.75) |
| Immune system disorders | Type iii immune complex mediated reaction | 11 | < 0.001 | 11.12(5.86–21.11) | 11.11(86.18) | 9.61(5.06) | 2.42(1.53) |
| Infections and infestations | Pertussis | 10 | < 0.001 | 18.73(9.25–37.92) | 18.72(129.60) | 14.69(7.26) | 2.65(1.68) |
| Nervous system disorders | Consciousness fluctuating | 8 | < 0.001 | 5.42(2.63–11.16) | 5.42(26.55) | 5.07(2.46) | 1.72(0.71) |
| Nervous system disorders | Tonic clonic movements | 7 | < 0.001 | 5.37(2.48–11.62) | 5.37(22.94) | 5.03(2.32) | 1.65(0.57) |
| Infections and infestations | Vaccination site cellulitis | 7 | < 0.001 | 4.55(2.11–9.79) | 4.55(18.07) | 4.31(2.00) | 1.51(0.45) |
| Psychiatric disorders | Posturing | 6 | < 0.001 | 10.05(4.25–23.79) | 10.05(42.22) | 8.81(3.73) | 1.96(0.77) |
| Musculoskeletal and connective tissue disorders | Bursal fluid accumulation | 5 | < 0.001 | 21.22(7.71–58.40) | 21.21(72.23) | 16.16(5.87) | 2.09(0.73) |
| Investigations | Csf white blood cell count negative | 5 | < 0.001 | 5.58(2.24–13.93) | 5.58(17.29) | 5.21(2.09) | 1.49(0.23) |
| Nervous system disorders | Opisthotonus | 5 | < 0.001 | 6.12(2.44–15.33) | 6.12(19.54) | 5.67(2.26) | 1.55(0.29) |
| Respiratory, thoracic and mediastinal disorders | Snoring | 5 | < 0.001 | 6.92(2.75–17.42) | 6.92(22.83) | 6.34(2.52) | 1.62(0.36) |
| Surgical and medical procedures | Supine position | 5 | < 0.001 | 22.74(8.19–63.14) | 22.73(76.53) | 17.01(6.13) | 2.11(0.74) |
| Investigations | Tuberculin test | 5 | < 0.001 | 6.63(2.64–16.66) | 6.63(21.65) | 6.10(2.43) | 1.60(0.33) |
| Infections and infestations | Abscess limb | 4 | < 0.001 | 6.53(2.33–18.27) | 6.53(16.98) | 6.01(2.15) | 1.44(0.04) |
| Nervous system disorders | Hyporeflexia | 4 | < 0.001 | 7.96(2.81–22.50) | 7.95(21.62) | 7.18(2.54) | 1.54(0.13) |
| Product issues | Product distribution issue | 4 | < 0.001 | 8.78(3.09–24.98) | 8.78(24.23) | 7.83(2.75) | 1.58(0.17) |
| Immune system disorders | Rubber sensitivity | 4 | < 0.001 | 12.73(4.35–37.25) | 12.73(36.02) | 10.77(3.68) | 1.73(0.28) |
| Nervous system disorders | Tonic convulsion | 4 | < 0.001 | 9.43(3.30–26.96) | 9.43(26.25) | 8.34(2.92) | 1.62(0.19) |

Tdap, tetanus, diphtheria, and acellular pertussis; PT, preferred term; SOC, System Organ Class; a, the number of reports; ROR, reporting odds ratio; CI, confidence interval; PRR, proportional reporting ratio; χ2, Chi-squared; EBGM, empirical Bayesian geometric mean; EBGM05, the lower 95 two-sided CI of EBGM; IC, information component; IC025, the lower limit of the 95% two-sided CI of the IC.

# Table S11. Signal strength of Tdap vaccine-related positive PT signals and their corresponding SOCs in pregnant females.

| SOC | PT | a | p–adjust | ROR(95%CI) | PRR(X²) | EBGM(EBGM05) | IC(IC025) |
| --- | --- | --- | --- | --- | --- | --- | --- |
| General disorders and administration site conditions | No adverse event | 212 | < 0.001 | 6.91(5.77–8.27) | 5.22(572.40) | 4.11(3.43) | 2.01(1.78) |
| Injury, poisoning and procedural complications | Product storage error | 47 | < 0.001 | 19.65(12.68–30.43) | 18.47(344.56) | 8.69(5.61) | 2.88(2.36) |
| General disorders and administration site conditions | Injection site swelling | 46 | < 0.001 | 4.66(3.32–6.53) | 4.43(95.23) | 3.63(2.59) | 1.76(1.29) |
| Injury, poisoning and procedural complications | Extra dose administered | 45 | < 0.001 | 10.82(7.33–15.96) | 10.22(221.94) | 6.42(4.35) | 2.50(1.99) |
| General disorders and administration site conditions | Injection site warmth | 27 | < 0.001 | 4.64(3.00–7.20) | 4.51(56.89) | 3.68(2.37) | 1.72(1.11) |
| General disorders and administration site conditions | Injected limb mobility decreased | 25 | < 0.001 | 13.4(7.77–23.10) | 12.98(146.73) | 7.33(4.25) | 2.52(1.84) |
| Injury, poisoning and procedural complications | Product administered at inappropriate site | 15 | < 0.001 | 14.82(7.22–30.43) | 14.54(94.80) | 7.77(3.78) | 2.40(1.51) |
| General disorders and administration site conditions | Injection site induration | 11 | < 0.001 | 5.22(2.61–10.43) | 5.16(27.32) | 4.07(2.04) | 1.63(0.68) |
| Injury, poisoning and procedural complications | Incorrect route of product administration | 10 | < 0.001 | 18.41(7.24–46.78) | 18.17(72.24) | 8.63(3.40) | 2.28(1.18) |
| Injury, poisoning and procedural complications | Medication error | 6 | < 0.001 | 17.58(5.35–57.74) | 17.45(42.32) | 8.48(2.58) | 1.94(0.55) |
| Pregnancy, puerperium and perinatal conditions | Neonatal disorder | 6 | < 0.001 | 10.98(3.80–31.74) | 10.9(30.88) | 6.66(2.30) | 1.78(0.44) |

Tdap, tetanus, diphtheria, and acellular pertussis; PT, preferred term; SOC, System Organ Class; a, the number of reports; ROR, reporting odds ratio; CI, confidence interval; PRR, proportional reporting ratio; χ2, Chi-squared; EBGM, empirical Bayesian geometric mean; EBGM05, the lower 95 two-sided CI of EBGM; IC, information component; IC025, the lower limit of the 95% two-sided CI of the IC.

# Table S12. Signal strength of Tdap vaccine-related positive PT signals and their corresponding SOCs in complete-case sensitivity analysis among non-pregnant females.

| SOC | PT | a | p–adjust | ROR(95%CI) | PRR(X²) | EBGM(EBGM05) | IC(IC025) |
| --- | --- | --- | --- | --- | --- | --- | --- |
| General disorders and administration site conditions | Injection site erythema | 1660 | < 0.001 | 2.38(2.26–2.51) | 2.16(1080.27) | 2.12(2.01) | 1.08(1.01) |
| General disorders and administration site conditions | Injection site swelling | 1346 | < 0.001 | 2.38(2.25–2.52) | 2.20(906.12) | 2.16(2.04) | 1.11(1.03) |
| Nervous system disorders | Syncope | 639 | < 0.001 | 3.51(3.24–3.82) | 3.36(1023.68) | 3.24(2.98) | 1.69(1.57) |
| Skin and subcutaneous tissue disorders | Skin warm | 522 | < 0.001 | 2.31(2.11–2.53) | 2.24(355.74) | 2.20(2.01) | 1.13(1.00) |
| Nervous system disorders | Loss of consciousness | 421 | < 0.001 | 3.19(2.88–3.52) | 3.10(577.44) | 3.00(2.71) | 1.58(1.43) |
| Injury, poisoning and procedural complications | Wrong product administered | 392 | < 0.001 | 5.65(5.08–6.27) | 5.47(1325.35) | 5.11(4.60) | 2.34(2.18) |
| Vascular disorders | Pallor | 382 | < 0.001 | 5.54(4.98–6.16) | 5.37(1258.26) | 5.02(4.51) | 2.31(2.16) |
| General disorders and administration site conditions | Injection site induration | 327 | < 0.001 | 2.71(2.42–3.03) | 2.66(327.37) | 2.59(2.31) | 1.36(1.20) |
| General disorders and administration site conditions | Injected limb mobility decreased | 271 | < 0.001 | 3.32(2.94–3.76) | 3.26(407.10) | 3.15(2.78) | 1.64(1.46) |
| Injury, poisoning and procedural complications | Fall | 223 | < 0.001 | 2.49(2.17–2.85) | 2.46(187.03) | 2.40(2.10) | 1.25(1.06) |
| Infections and infestations | Cellulitis | 178 | < 0.001 | 3.33(2.86–3.87) | 3.29(270.32) | 3.17(2.72) | 1.64(1.42) |
| General disorders and administration site conditions | Injection site mass | 150 | < 0.001 | 2.45(2.08–2.89) | 2.43(122.55) | 2.38(2.02) | 1.23(1.00) |
| General disorders and administration site conditions | Injection site nodule | 135 | < 0.001 | 4.48(3.76–5.34) | 4.43(335.87) | 4.20(3.53) | 2.03(1.78) |
| Musculoskeletal and connective tissue disorders | Musculoskeletal pain | 127 | < 0.001 | 3.28(2.74–3.92) | 3.25(188.43) | 3.14(2.62) | 1.62(1.36) |
| General disorders and administration site conditions | Induration | 125 | < 0.001 | 2.57(2.15–3.08) | 2.55(113.88) | 2.49(2.08) | 1.29(1.03) |
| Musculoskeletal and connective tissue disorders | Joint range of motion decreased | 108 | < 0.001 | 2.65(2.18–3.21) | 2.63(105.11) | 2.56(2.11) | 1.33(1.05) |
| Injury, poisoning and procedural complications | Head injury | 103 | < 0.001 | 3.96(3.24–4.84) | 3.93(211.98) | 3.75(3.07) | 1.86(1.58) |
| Nervous system disorders | Presyncope | 97 | < 0.001 | 2.55(2.08–3.13) | 2.54(86.98) | 2.47(2.02) | 1.28(0.98) |
| General disorders and administration site conditions | Local reaction | 90 | < 0.001 | 4.42(3.57–5.48) | 4.39(220.45) | 4.17(3.36) | 2.00(1.69) |
| Injury, poisoning and procedural complications | Product administered at inappropriate site | 85 | < 0.001 | 3.05(2.45–3.80) | 3.04(110.85) | 2.94(2.36) | 1.51(1.20) |
| Eye disorders | Eye movement disorder | 79 | < 0.001 | 4.96(3.94–6.24) | 4.93(229.35) | 4.64(3.68) | 2.14(1.81) |
| General disorders and administration site conditions | Nodule | 79 | < 0.001 | 3.11(2.48–3.90) | 3.09(106.90) | 2.99(2.39) | 1.54(1.21) |
| Nervous system disorders | Dyskinesia | 75 | < 0.001 | 3.38(2.67–4.26) | 3.36(118.16) | 3.24(2.56) | 1.64(1.31) |
| Infections and infestations | Injection site cellulitis | 70 | < 0.001 | 3.88(3.05–4.95) | 3.86(140.06) | 3.69(2.90) | 1.82(1.47) |
| Musculoskeletal and connective tissue disorders | Posture abnormal | 63 | < 0.001 | 7.90(6.07–10.28) | 7.86(334.67) | 7.08(5.44) | 2.68(2.31) |
| Injury, poisoning and procedural complications | Medication error | 49 | < 0.001 | 4.45(3.33–5.95) | 4.44(121.77) | 4.20(3.14) | 1.97(1.55) |
| General disorders and administration site conditions | Injection site oedema | 44 | < 0.001 | 4.92(3.62–6.69) | 4.90(126.67) | 4.61(3.39) | 2.08(1.64) |
| Nervous system disorders | Seizure like phenomena | 36 | < 0.001 | 4.16(2.97–5.84) | 4.15(80.81) | 3.95(2.82) | 1.85(1.37) |
| Musculoskeletal and connective tissue disorders | Bursitis | 33 | < 0.001 | 2.99(2.11–4.24) | 2.98(41.54) | 2.89(2.04) | 1.43(0.93) |
| Investigations | X-ray limb | 31 | < 0.001 | 3.03(2.11–4.34) | 3.02(39.97) | 2.93(2.04) | 1.44(0.92) |
| Musculoskeletal and connective tissue disorders | Muscle rigidity | 29 | < 0.001 | 6.86(4.67–10.08) | 6.85(130.32) | 6.26(4.26) | 2.39(1.84) |
| Investigations | X-ray limb normal | 27 | < 0.001 | 4.00(2.71–5.91) | 3.99(56.93) | 3.81(2.58) | 1.76(1.21) |
| Nervous system disorders | Hypotonia | 26 | < 0.001 | 3.32(2.23–4.92) | 3.31(39.83) | 3.19(2.15) | 1.53(0.97) |
| Musculoskeletal and connective tissue disorders | Limb mass | 25 | < 0.001 | 3.11(2.08–4.64) | 3.10(33.89) | 3.00(2.01) | 1.45(0.87) |
| Musculoskeletal and connective tissue disorders | Rotator cuff syndrome | 25 | < 0.001 | 3.48(2.32–5.21) | 3.47(41.67) | 3.34(2.23) | 1.59(1.01) |
| Respiratory, thoracic and mediastinal disorders | Pharyngeal oedema | 24 | < 0.001 | 4.07(2.69–6.15) | 4.06(51.92) | 3.87(2.56) | 1.76(1.18) |
| Nervous system disorders | Generalised tonic-clonic seizure | 22 | < 0.001 | 3.42(2.22–5.25) | 3.41(35.53) | 3.28(2.14) | 1.55(0.93) |
| Nervous system disorders | Altered state of consciousness | 20 | < 0.001 | 4.68(2.97–7.37) | 4.67(53.59) | 4.41(2.80) | 1.89(1.24) |
| Eye disorders | Mydriasis | 20 | < 0.001 | 3.92(2.49–6.16) | 3.91(40.74) | 3.74(2.38) | 1.69(1.04) |
| Injury, poisoning and procedural complications | Face injury | 19 | < 0.001 | 4.85(3.04–7.73) | 4.84(53.67) | 4.56(2.86) | 1.91(1.25) |
| Infections and infestations | Injection site infection | 18 | < 0.001 | 3.50(2.18–5.63) | 3.50(30.37) | 3.36(2.09) | 1.54(0.86) |
| Eye disorders | Eyelid oedema | 17 | < 0.001 | 5.25(3.20–8.62) | 5.24(53.81) | 4.91(2.99) | 1.97(1.27) |
| General disorders and administration site conditions | Injection site joint pain | 17 | < 0.001 | 4.95(3.02–8.12) | 4.95(49.54) | 4.65(2.84) | 1.91(1.21) |
| Injury, poisoning and procedural complications | Skin laceration | 16 | < 0.001 | 3.80(2.29–6.29) | 3.79(30.99) | 3.63(2.19) | 1.61(0.89) |
| Infections and infestations | Rash pustular | 15 | < 0.001 | 3.94(2.34–6.64) | 3.93(30.87) | 3.76(2.23) | 1.63(0.89) |
| Nervous system disorders | Hyporesponsive to stimuli | 14 | < 0.001 | 5.25(3.04–9.06) | 5.24(44.27) | 4.91(2.84) | 1.91(1.14) |
| Investigations | Neurological examination normal | 13 | < 0.001 | 3.66(2.09–6.41) | 3.66(23.73) | 3.51(2.01) | 1.52(0.73) |
| General disorders and administration site conditions | Injection site discharge | 11 | < 0.001 | 4.45(2.41–8.20) | 4.44(27.37) | 4.21(2.28) | 1.67(0.81) |
| Nervous system disorders | Radiculitis brachial | 11 | < 0.001 | 15.36(7.93–29.75) | 15.35(118.03) | 12.48(6.44) | 2.61(1.70) |
| Nervous system disorders | Slow response to stimuli | 11 | < 0.001 | 7.51(4.01–14.05) | 7.50(55.25) | 6.79(3.63) | 2.13(1.25) |
| Immune system disorders | Type iii immune complex mediated reaction | 10 | < 0.001 | 11.17(5.69–21.92) | 11.16(78.28) | 9.60(4.89) | 2.36(1.42) |
| Nervous system disorders | Consciousness fluctuating | 8 | < 0.001 | 5.23(2.54–10.76) | 5.22(25.19) | 4.89(2.38) | 1.69(0.68) |
| Nervous system disorders | Tonic clonic movements | 7 | < 0.001 | 5.58(2.57–12.11) | 5.58(24.13) | 5.20(2.40) | 1.68(0.60) |
| Infections and infestations | Vaccination site cellulitis | 7 | < 0.001 | 6.32(2.90–13.77) | 6.32(28.42) | 5.82(2.67) | 1.77(0.69) |
| Psychiatric disorders | Posturing | 6 | < 0.001 | 10.24(4.31–24.30) | 10.23(42.83) | 8.91(3.75) | 1.96(0.77) |
| Musculoskeletal and connective tissue disorders | Bursal fluid accumulation | 5 | < 0.001 | 23.62(8.42–66.27) | 23.61(78.19) | 17.33(6.18) | 2.12(0.74) |
| Investigations | Csf white blood cell count negative | 5 | < 0.001 | 6.14(2.45–15.40) | 6.14(19.55) | 5.67(2.26) | 1.55(0.29) |
| Nervous system disorders | Opisthotonus | 5 | < 0.001 | 6.27(2.50–15.73) | 6.26(20.07) | 5.78(2.30) | 1.56(0.30) |
| Respiratory, thoracic and mediastinal disorders | Snoring | 5 | < 0.001 | 7.14(2.83–18.03) | 7.14(23.64) | 6.50(2.57) | 1.64(0.37) |
| Surgical and medical procedures | Supine position | 5 | < 0.001 | 21.93(7.90–60.90) | 21.92(73.57) | 16.42(5.91) | 2.10(0.73) |
| Investigations | Tuberculin test | 5 | < 0.001 | 6.98(2.77–17.60) | 6.98(22.98) | 6.37(2.52) | 1.63(0.36) |
| Infections and infestations | Abscess limb | 4 | < 0.001 | 6.64(2.37–18.63) | 6.64(17.28) | 6.09(2.17) | 1.45(0.05) |
| Injury, poisoning and procedural complications | Exposure during pregnancy | 4 | < 0.001 | 10.23(3.55–29.50) | 10.23(28.56) | 8.91(3.09) | 1.65(0.22) |
| Infections and infestations | Pertussis | 4 | < 0.001 | 12.28(4.20–35.94) | 12.28(34.53) | 10.40(3.55) | 1.72(0.27) |
| Nervous system disorders | Tonic convulsion | 4 | < 0.001 | 9.10(3.18–26.00) | 9.09(25.10) | 8.05(2.82) | 1.60(0.18) |

Tdap, tetanus, diphtheria, and acellular pertussis; PT, preferred term; SOC, System Organ Class; a, the number of reports; ROR, reporting odds ratio; CI, confidence interval; PRR, proportional reporting ratio; χ2, Chi-squared; EBGM, empirical Bayesian geometric mean; EBGM05, the lower 95 two-sided CI of EBGM; IC, information component; IC025, the lower limit of the 95% two-sided CI of the IC.

# Table S13. Single-vaccine Tdap sensitivity analysis for the signal of **radiculitis brachial and bursal fluid accumulation** in non-pregnant females, opisthotonus in the adolescents .

| Subgroup | PT | a | p-adjust | ROR(95%CI) | PRR(X²) | EBGM(EBGM05) | IC(IC025) |
| --- | --- | --- | --- | --- | --- | --- | --- |
| Non-pregnant females | Radiculitis brachial | 12 | < 0.001 | 27.87(14.89–52.19) | 27.82(252.98) | 22.87(12.21) | 3.04(2.17) |
| Non-pregnant females | Bursal fluid accumulation | 4 | < 0.001 | 35.13(11.56–106.75) | 35.10(103.08) | 27.52(9.06) | 2.00(0.52) |
| Adolescents | Opisthotonus | 2 | < 0.001 | 20.34(4.10–100.90) | 20.31(27.54) | 15.48(3.12) | 1.22(-0.81) |

Tdap, tetanus, diphtheria, and acellular pertussis; PT, preferred term; a, the number of reports; ROR, reporting odds ratio; CI, confidence interval; PRR, proportional reporting ratio; χ2, Chi-squared; EBGM, empirical Bayesian geometric mean; EBGM05, the lower 95 two-sided CI of EBGM; IC, information component; IC025, the lower limit of the 95% two-sided CI of the IC.

# Table S14. Case-deletion analysis for the signal of **radiculitis brachial** following Tdap vaccination in non-pregnant females.

| Cases Removed (k) | Remaining cases (a－k) | p–adjust | ROR(95%CI) | PRR(X²) | EBGM(EBGM05) | IC(IC025) |
| --- | --- | --- | --- | --- | --- | --- |
| 0 (Baseline) | 12 | < 0.001 | 14.42(7.71–27.00) | 14.41(122.10) | 11.93(6.38) | 2.64(1.77) |
| 1 | 11 | < 0.001 | 13.22(6.90–25.32) | 13.21(102.80) | 11.11(5.80) | 2.53(1.63) |
| 2 | 10 | < 0.001 | 12.02(6.11–23.63) | 12.01(84.91) | 10.26(5.22) | 2.41(1.47) |
| 3 | 9 | < 0.001 | 10.82(5.33–21.93) | 10.81(68.49) | 9.39(4.63) | 2.28(1.29) |
| 4 | 8 | < 0.001 | 9.61(4.57–20.23) | 9.61(53.62) | 8.48(4.03) | 2.13(1.10) |
| 5 | 7 | < 0.001 | 8.41(3.82–18.51) | 8.41(40.37) | 7.54(3.43) | 1.96(0.87) |
| 6 | 6 | < 0.001 | 7.21(3.10–16.78) | 7.21(28.82) | 6.58(2.83) | 1.77(0.60) |
| 7 | 5 | < 0.001 | 6.01(2.40–15.04) | 6.01(19.07) | 5.58(2.23) | 1.54(0.28) |
| 8 | 4 | < 0.001 | 4.81(1.74–13.29) | 4.81(11.21) | 4.54(1.64) | 1.26(-0.12) |

Tdap, tetanus, diphtheria, and acellular pertussis; k, the number of removed reports; a, the number of total reports; ROR, reporting odds ratio; CI, confidence interval; PRR, proportional reporting ratio; χ2, Chi-squared; EBGM, empirical Bayesian geometric mean; EBGM05, the lower 95 two-sided CI of EBGM; IC, information component; IC025, the lower limit of the 95% two-sided CI of the IC.

# Table S15. Restricted time-window disproportionality analysis for the signal of radiculitis brachial following Tdap vaccination in non-pregnant females.

| SOC | PT | a | p-adjust | ROR(95%CI) | PRR(X²) | EBGM(EBGM05) | IC(IC025) |
| --- | --- | --- | --- | --- | --- | --- | --- |
| Nervous system disorders | Radiculitis brachial | 11 | < 0.001 | 15.23(7.83–29.64) | 15.22(115.20) | 12.21(6.27) | 2.60(1.68) |

Tdap, tetanus, diphtheria, and acellular pertussis; PT, preferred term; SOC, System Organ Class; a, the number of reports; ROR, reporting odds ratio; CI, confidence interval; PRR, proportional reporting ratio; χ2, Chi-squared; EBGM, empirical Bayesian geometric mean; EBGM05, the lower 95 two-sided CI of EBGM; IC, information component; IC025, the lower limit of the 95% two-sided CI of the IC.
